# An orderly single-trial organization of population dynamics in premotor cortex predicts behavioral variability

**DOI:** 10.1038/s41467-018-08141-6

**Published:** 2019-01-15

**Authors:** Ziqiang Wei, Hidehiko Inagaki, Nuo Li, Karel Svoboda, Shaul Druckmann

**Affiliations:** 1 Janelia Research Campus, HHMI, Ashburn, VA 20147 USA; 2 The Solomon H. Snyder Department of Neuroscience, Johns Hopkins University School of Medicine, Baltimore, MD 21205 USA; 3 Department of Neuroscience, Baylor College of Medicine, Houston, TX 77030 USA; 4 Department of Neurobiology, Stanford University, Stanford, CA 94304 USA

## Abstract

Animals are not simple input-output machines. Their responses to even very similar stimuli are variable. A key, long-standing question in neuroscience is to understand the neural correlates of such behavioral variability. To reveal these correlates, behavior and neural population activity must be related to one another on single trials. Such analysis is challenging due to the dynamical nature of brain function (e.g., in decision making), heterogeneity across neurons and limited sampling of the relevant neural population. By analyzing population recordings from mouse frontal cortex in perceptual decision-making tasks, we show that an analysis approach tailored to the coarse grain features of the dynamics is able to reveal previously unrecognized structure in the organization of population activity. This structure is similar on error and correct trials, suggesting dynamics that may be constrained by the underlying circuitry, is able to predict multiple aspects of behavioral variability and reveals long time-scale modulation of population activity.

## Abstract (web summary)

To explain the neural correlates of behavior and its variability, one must analyze single-trial population dynamics. Here, the authors develop a statistical method that extracts low-dimensional dynamics that explain behavior better than high-dimensional neural activity revealing unexpected structure.

## Introduction

Decision making is a central behavioral paradigm in neuroscience. In such tasks, animals sample sensory inputs, generate a behavioral choice, hold it in memory, prepare and execute an action, and finally compare the outcome to the expected delivery of reward. These elaborate behavioral computations manifest in complex neural dynamics, and occur across multiple temporal scales and brain areas^1–11^. For example, recent studies in mice identified anterior lateral motor (ALM) cortex as a critical circuit node for perceptual decision tasks^9–12^. ALM neurons exhibit complex, heterogeneous, epoch-dependent dynamics; population dynamics in ALM and connected thalamic nuclei^13^ evolve over time scales of milliseconds to seconds. These neural dynamics reflect the confluence of sensory information with representation of upcoming movement and other internal states. Yet the complexity of population dynamics and experimental limitations combine to render the structure of ALM population activity and how it encodes behavioral variables and internal states.

Across trials, identical stimuli give rise to variable neural responses and behavior. Hence, averaging trials obscures the link between behavioral and neural variability. Neural activity may explain variable behavioral outcomes, yet variability in single neurons is challenging to analyze because spikes are noisy. Moreover, simultaneous recordings have consistently revealed diverse neural activity, both within and among brain areas, complicating efforts to relate neural dynamics to underlying computations and to behavior. Methods that analyze population activity can leverage simultaneous recordings to pool information across neurons, revealing information that is difficult to decode directly from single neurons^8,14–27^. Typically, these approaches infer intermediary variables (latent variables) from the activity of simultaneously-recorded neurons in single trials, then use these variables as the basis to predict behavioral outputs. Such methods have improved estimation in brain-computer interfaces^28,29^ and predicted behavioral properties such as reaction time^7^, indecision and hesitation^23^.

In this study, we develop a variant of latent space dynamical system (LDS) models to uncover information from complex, temporally heterogenous neural recordings. We assumed population activity in single trials can be explained by the evolution in time of a lower dimensional dynamical system learned from the data. The state of these learned dynamics represents shared activity across neurons^30^. We leverage the conceptual and computational simplicity of LDS models but extend their efficacy by allowing the underlying dynamics to change over time. Importantly, switches between distinct dynamical systems occur at cued times corresponding to the transitions between trial epochs when population dynamics are known to change^9–11^. This approach represents a compromise between two extremes: assuming the underlying population dynamics are linear and fixed across time^14–17,24–26^ versus using nonlinear dynamical models that allow switches between sets of linear dynamics at any given time^18,27,31^. Striking a middle ground is important. Static models fail to capture the complexity of neural dynamics, whereas dynamical models that require tracking of combinatorial numbers of past switching options, are difficult to fit.

We reveal previously unrecognized structure within the population dynamics of ALM during a two-alternative-forced choice task. In this task, mice presented with one of two sensory stimuli during a sample period were required to execute a specific action matched to each sensory stimulus, but only after a delay, during the response period.

We analyzed simultaneous recordings from ALM and found that our LDS models captured a large fraction of neural variability. The low-dimensional latent variables in our model, referred to as the shared activity space (SAS), reveal dynamics that are robustly maintained for seconds pre-decision and post-decision, far longer than that was required to successfully perform the task i.e., just bridging between the sample and response periods. SAS dynamics predicted multiple forms of single-trial behavioral variability, such as response latency and correctness of animal responses. Latent dynamics discovered during correct trials also illuminated activity during error trials: SAS dynamics predicted an animal’s error hundreds of milliseconds to seconds before the actual action occurred.

Our results have implications for understanding the circuits underlying slow dynamics that are associated with decision making and short-term memory. First, we find that dynamics are consistent across long timescales: within-epoch dynamics repeated reliably across multiple consecutive trials (timescales of minutes), demonstrating where and how long-timescale internal states can be found in the brain. Indeed, we can infer longer timescale behavioral measures, e.g., reward on a previous trial, from our model. Second, we find population activity maintains the rank order of individual trials across epochs within a trial type, inconsistent with certain types of attractor-based models^32^.

In summary, we find that modes of shared population activity reveal novel properties of circuit dynamics, allow more accurate descriptions of the single-trial variability of dynamics, and can yield useful quantitative measures of the neural substrate of behavior both in single trials and on longer timescales.

## Results

### Epoch-dependent dynamics in anterior lateral motor cortex

**Fig. 1:**
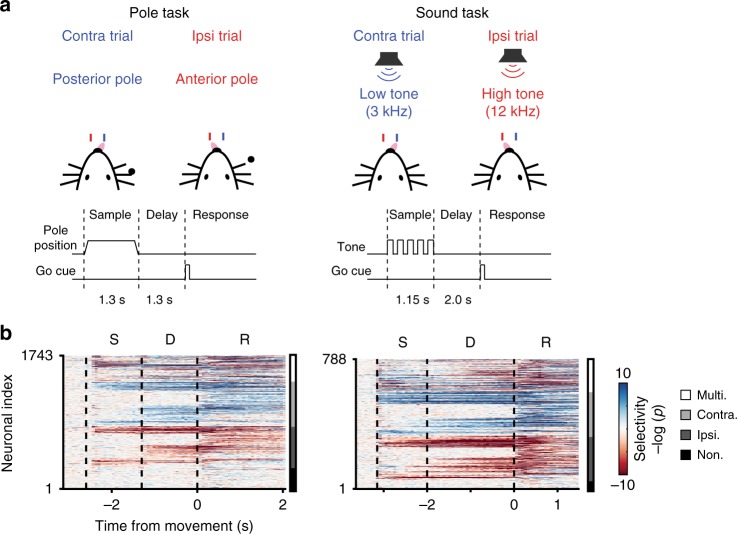
Neural activity of anterior lateral motor cortex neurons exhibits complex, heterogenous activity. **a** Schematic description of experiments. Mice were trained to report pole position (left) or frequency of tone (right) by directional licking, following a delay period after the end of stimulus presentation (contra trial, lick right; ipsi trial, lick left). **b** Heatmap of instantaneous trial-type selectivity, displayed as negative log of *p*-value (two-sample *t*-test), (blue: contra-preferring; red: ipsi-preferring). For visual clarity neurons were sorted by response category, shown in bar on right (black, non-selective; dark gray, ipsi-monophasic selective; light gray, contra-monophasic selective; white, multiphasic selective, where neurons show strong switches of selectivity during a task)

Multiple studies have established a causal link between ALM preparatory activity and movement^9–12^. Here, we analyzed electrophysiological recordings from mice performing a delayed response task. In one paradigm, mice (*n* = 33) reported the position of a pole (anterior or posterior) by directional licking (lick-left or lick-right). Mice were allowed to respond after a 1.3-s delay, when an auditory cue signaled the onset of the response epoch. In the second paradigm, mice (*n* = 6) reported the frequency (pitch) of an auditory cue after a 2-s delay (Fig. 1a).

ALM neurons exhibit complex, heterogeneous dynamics. Consistent with previous studies, we observed a large proportion of ALM neurons exhibited persistent and ramping preparatory activity during the delay epoch before the movement^9–12^. This was true across multiple levels: single-neuron or population activity; trial-type decoding based on single-neurons, which changed substantially over time for many neurons and at the level of population selectivity. We defined instantaneous neuronal selectivity as the differences between the responses, at a specific time point, in lick-left versus lick-right trials. Tracking instantaneous activity across the length of the trial, we find >50% of ALM neurons (*n* = 917/1743, pole task; *n* = 444/788, sound task) changed their selectivity from the delay to the response epochs. Neurons became selective at various time points during the trial, with many changes appearing at the onset of epochs (Fig. 1b). At the population level, we found strong switches in dynamics^27^ when analyzing simultaneous population activity (6–31 units per session, 55 sessions; Supplementary Table 1). Defining population trial-type selectivity by linear discriminant analysis (LDA) decoders at separate times, we found that decoders were stable within epochs, but diverged across epochs. These observations held true for both tasks and even when neurons were combined across sessions into a pseudo-population (Supplementary Fig. 1).

### Neural dynamics and the shared-activity space

Such complex neural dynamics are difficult to interpret at the level of raw population activity (i.e., a set of time series representing spikes). We analyzed population activity through latent dynamical system models (LDS) that directly model the population activity^8,14–27,31^ effectively as a combination of a small number of shared patterns of neurons, with a compromise between static LDS models that do not capture the complexity of ALM activity and fully switching linear dynamical system models (sLDS), which suffer from combinatorial difficulties in fits. Hence, we instead assumed static linear dynamics during each behavioral epoch but allowed dynamics to change at cued times, i.e., switches of behavioral epochs:

1 $${\boldsymbol{r}}\left( t \right) = {\boldsymbol{W}}^{{\boldsymbol{proj}}}\left( s \right){\boldsymbol{x}}\left( t \right) + {\boldsymbol{r}}_0 + {\boldsymbol{v}}\left( t \right)$$

2 $${\boldsymbol{x}}\left( t \right) = {\boldsymbol{W}}^{{\boldsymbol{mode}}}\left( s \right){\boldsymbol{x}}\left( {t - 1} \right) + {\boldsymbol{w}}\left( {t - 1} \right)$$

**Fig. 2:**
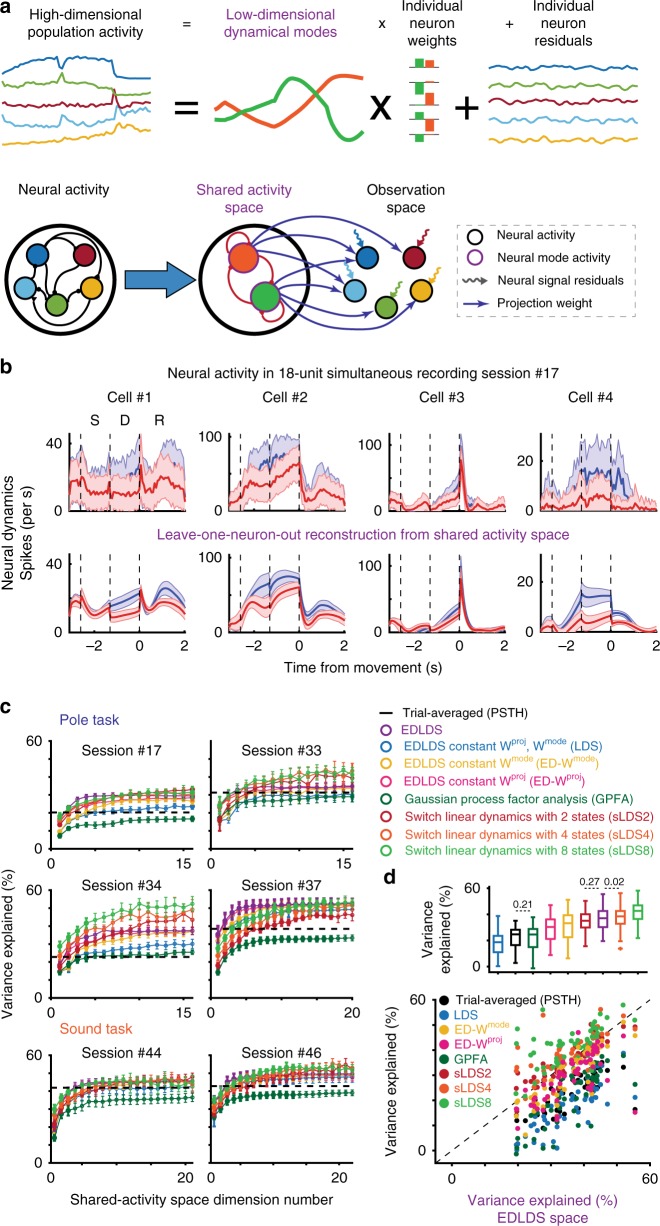
Latent dynamical system modeling of neural activity and the shared-activity space. **a** Schematic description of time-varying linear dynamical systems model. Top: population activity is decomposed into a few activity modes shared across neurons. The activity of each neuron is described as a weighted sum of the shared modes and a single neuron residual activity. Bottom: alternative representation of model as a two-layer neural network. **b** Model reproduces neural activity in a leave-one-neuron-out scenario; the activity of a given neuron is estimated from the dynamics of the other simultaneously recorded neurons. Top: Observed neuronal activity of four neurons randomly picked from Session #17, (ipsi trials red, contra trials blue). Bottom: prediction of neuronal activity on prediction data where the activity of that neuron is unobserved. Shaded area, std. across trials. **c** Comparison of shared activity space fits for different models: event-dependent dynamical systems (EDLDS, purple, our model); three variations of our model—constant dynamical systems (LDS, blue), constant ***W***^**mode**^ (yellow) and constant ***W***^**proj**^ (pink); two simpler models—PSTH (mean activity in time for each trial type; black) and Gaussian process factor analysis (GPFA, dark green); and three variations of switching linear dynamical systems with 2 (red), 4 (orange) and 8 (light green) hidden states as a function of number of latent dimensionalities for 6 recording sessions (4 from pole tasks, 2 from sound tasks, note all data from simultaneous recordings, not pseudo-populations). **d** Comparison of variance explained for different models for all recording sessions (*n* = 55). Scatter plot below shows each single session as a dot; boxplot (center line, median; bounds of box, 25 and 75%; whiskers, 1.5 interquartile range) above, shows across-session statistical summary; *p* < .001 for paired-comparisons (sign-rank test) between two models in boxplots without explicitly values

We assume that neural population activity, the full-neural space (FNS), ${\boldsymbol{r}}\left( t \right) \in {\boldsymbol{R}}^N$, can be modeled as a weighted sum of neural modes, ${\boldsymbol{x}}\left( t \right) \in {\boldsymbol{R}}^M\left( {M < N} \right)$, i.e., a low-dimensional latent space, through a projection matrix ***W***^***proj***^. We refer to this latent space of **x** as the shared activity space (SAS) since each latent mode represents common dynamics shared by many neurons. The model considers the difference between the observed activity in the FNS and the activity explained by the SAS as neural residuals, ${\boldsymbol{v}}({\boldsymbol{t}}) \in {\boldsymbol{R}}^N$, that are independent for each neuron (Fig. 2a, top). This model can be represented as a two-layer network: The first layer, corresponding to the SAS, is represented by a set of implicit units with layer-internal dynamics, while the second layer comprises units whose activation represents the measured population activity in FNS. The activity of the second layer is the projection up of the SAS by ***W***^***pro****j*^ and the addition of the neural residuals (Fig. 2a, bottom). In SAS, temporal dynamics and interaction of latent modes are modeled as an LDS expressed by the matrix ***W***^***mode***^ that incorporates a stochastic term $({\boldsymbol{t}}) \in {\boldsymbol{R}}^M$, which varies across time and trials. This term can represent input from neurons in other parts of the brain, such as sensory areas, and is often referred to as an innovation term. Notably, both matrices that describe the dynamics, ***W***^***proj***^ and ***W***^***mode***^, are epoch-dependent (indexed by s). More generally, for cases without explicit epoch-design, switches can be linked to other known events, such as shifts in spatial location or changes in the environment. We refer to these models as event-dependent linear dynamical systems models (EDLDS). For the delayed response task, the transitions between the epochs in each trial are the only events that drive switches.

For a model fit to a set of population activity, we measured the goodness of fit (R^2^) using a leave-one-neuron-out (LONO) approach^15^, predicting the activity of a particular neuron when it is left out of the model fitting. If the dynamics of individual neurons are correlated and neural-mode dynamics have been well-estimated, it should be possible to predict the activity of any neuron from the estimation of the neural mode dynamics derived from test trials in which the activity of that neuron was held out. Comparing the neural activity with its LONO estimation, we found EDLDS model explained a large fraction of variability (*R*^2^ = .31, Fig. 2b, Session #17, *N* = 18 neurons, *M* = 4 modes). For most sessions, LONO R^2^ initially increased with latent dimensionality and then saturated (Fig. 2c). We compared how much variance was explained by distinct configurations of our model, e.g., allowing only the latent dynamics to change from epoch to epoch versus allowing only the relation between latent dynamics and neural activity to change across epochs. We also compared our models to a large set of other models of varying complexity^14,15,31^. For a reference value, we compared all models to a prediction based on the trial-averaged response (i.e., a neuron’s peri-stimulus time histogram, PSTH, for a particular trial type).

Incorporating the epoch-dependent matrices ***W***^***proj***^ and ***W***^***mode***^ yielded better fits compared to holding either constant (Fig. 2c), consistent with the strong epoch-dependent change in dynamics. As expected, the EDLDS model outperformed simpler models (Fig. 2d, PSTH, Gaussian process factor analysis (GPFA), and LDS; *p* < .001) and performed only marginally worse than models that allowed switching at any time point (*p* = .27, sLDS2; *p* = .02, sLDS4; *p* < .001, sLDS8 and other models; sign-rank test). In our hands, the run time of sLDS models was 10–20 times longer; their fitting was more complex, conceptually and algorithmically, and there were hints of potential overfitting (i.e., switches at unexplained and inconsistent times; Supplementary Fig. 2cd). Thus, we confirmed the ESLDS model provided good fits to the population dynamics that are comparable to a true sLDS, despite its simplicity.

EDLDS models that provide good fits shared a number of properties: across sessions, models typically had a long time constant at the delay and the shortest one at the pre-sample epoch (Supplementary Fig. 2e). Both properties are consistent with our expectation of a delayed-response task. In addition, the approximate dimensionality of the SAS was similar across sessions, (3 or 4, pole task; 6, sound task; Supplementary Fig. 2b) despite the number of simultaneously recorded units spanning a fairly wide range (6–31 units per session, 55 sessions; Supplementary Table 1).

### Decoding behavioral variability from shared activity space

**Fig. 3:**
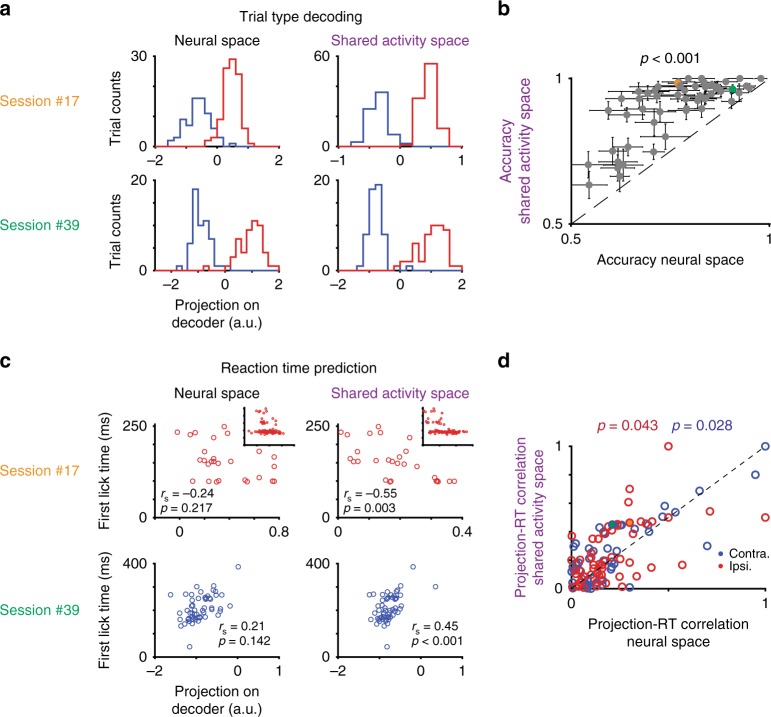
Neural dynamics in shared activity space correlates with trial-by-trial variability in behavioral reaction time and performance on single trials. **a** Histograms of projection of all correct trials on decoder separately for each trial type (ipsi trials red, contra trials blue), for full neural space (left) and shared activity space (right). Session #17, upper; Session #39, lower. **b** Accuracy of decoders based on full neural space (x-axis) and shared neural space (*y*-axis) across all sessions. Each circle represents a recording session. Error bar, std. across trials. Session #17, orange circle; Session #39, green circle. **c** Correlation between projection on decoder (*x*-axis) and reaction time to the first lick (*y*-axis) for example session for decoder based on full neural space (left) and shared neural space (right). Top: Session #17, ipsi. trial types (blue), long reaction time trials (>100 ms), in main plot and all trials in inset. Bottom: Session #39, contra trial type (red), same convention. **d** Scatter across all sessions of correlation between measured and decoded reaction time. Full neural space decoder correlation (*x*-axis) versus shared neural space decoder correlation (*y*-axis). For each session only one trial-type is considered. Each circle represents a session. Color of circle represents trial type; *p*-value presents significance that correlation is strong in SAS (sign-rank test). Session #17, ipsi. trial type, orange circle; Session #39, contra. trial type, green circle

Having extracted single-trial dynamics in the SAS, we measured their ability to decode behavioral variables. If the SAS judiciously pools neural activity across neurons and time, one expects better performance in decoding behavioral variability from the SAS than from the FNS. We first tested whether trial type could be decoded, training behavioral-choice decoders on either the FNS or the SAS at 300 ms before response onset. While both decoders successfully differentiated trial types (Fig. 3a), decoders running on the SAS were significantly more accurate (*p* < .001, sign-rank test, Fig. 3b; decoding based on different times within the delay period yielded qualitatively similar results).

Next, we considered more subtle predictions, i.e., explaining the variability in the timing of movement^7,33,34^. We performed this prediction based on the single-trial projection of population dynamics on behavioral-choice decoders. Projection values based on the SAS were more correlated with movement onset than those based on the FNS (Fig. 3c; top inner, Session #17, ipsi. trials; FNS, left, *r*_s_ = −.31; *p* = .001, sign-rank test; SAS, right, *r*_s_ = −.47; *p* < .001; bottom, Session #39, contra. trials; FNS, left, *r*_s_ = .21; p = .142; SAS, right, *r*_s_ = .45; *p* < .001; across sessions, Fig. 3d). We note that in multiple sessions, reactions were a mix of fast (100 ± 3 ms, mean ± std.) and slow responses (>200 ms; a long-tail distribution, Fig. 3c, top). We consider them separately; the longer reaction times were predicted accurately in the SAS (Fig. 3c, top; Session #09; reaction time >100 ms; FNS, left, *r*_s_ = −.24; *p* = .217; SAS, right, *r*_s_ = −.55; *p* = .003), but neither FNS nor SAS predicted fast-response trials well (*p* > 0.05, *r*_s_ = 0, sign-rank test). Comparing across latent space models, we found our ESLDS model outperformed most models in both trial type and reaction time decoding (Supplementary Fig. 3).

Inferior performance in FNS may be due to poor model fitting, since the larger dimensionality of FNS forces decoders based on it to have more parameters. Therefore, we experimented extensively with regularization of FNS, yet the EDLDS-SAS performance was superior across all regularization parameters and methods^35^ (Supplementary Fig. 4). Moreover, although other models had a similar dimensionality and thus did not require more regularization, these still yielded inferior performance to the EDLDS-SAS. Notably, all the information to make predictions was in the FNS; the EDLDS and other SAS models are fit without additional side information. These models make specific assumptions regarding an underlying model for the dynamics, however, which allows them to perform effective denoising of signals and thus yield better predictors^7^.

### Long time scale single-trial signals across epochs

Successful performance of the delayed task requires trial-type signals to propagate from the sample to the delay epoch. Accordingly we expect to find that trials of distinct types can be differentiated across this period. Beyond this minimal organization of population activity, it is not clear whether further levels of organization can be discerned. Such organization might reflect additional internal states that can be quantified, tracked, and potentially related to behavior^8,25^.

**Fig. 4:**
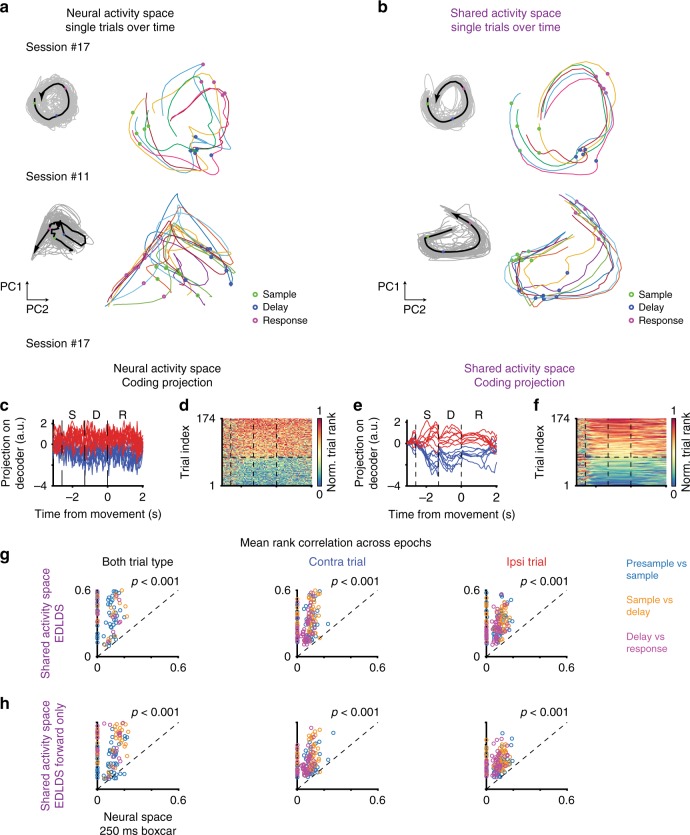
Maintenance of orderly internal representation of trial identity in the shared-activity space. **a**–**b** Neural dynamics spanned by first two principal components (contra trials, Session #17, top; contra trials, Session #11, bottom). Thin lines, single-trial dynamics; black thick lines, averaged dynamics across trials; circles, onsets of behavioral epochs: sample (green); delay (blue); response (red). **a** full neural space; **b** shared activity space. Full neural space dynamics shows sharp transitions from one behavioral epoch to the other and random order on single trials. Shared activity space dynamics show smooth transitions in time and strongly ordered dynamics of single trials, where the inner trials (relative to mean) stay inner across time and behavioral epochs. **c**–**f** Neural dynamics projected to instantaneous coding directions. **c** Traces of single trial projections to the trial-types coding direction in full neural space. **d** Rank of all trials across time. Data sorted according to average rank in sample-delay epochs. **e**–**f** The same as **c**–**d** except for those in shared activity space. **g** Scatter of temporal correlations of trial identity across adjacent behavioral epochs in full neural space (*x*-axis) and shared activity space (*y*-axis). Each dot represents a session, color indicates transition type: between pre-sample and sample epochs (blue); between sample and delay epochs (orange); between delay and response epochs (purple). **h** the same convention as **g** except for forward-only pass EDLDS fit. All *p* < .001, sign-rank test

SAS maintains a consistent relationship between trials from the same trial-type across time while FNS does not. Visualizing the projection of the first two principal components of the raw population activity across behavioral epochs, the trajectories of individual trials cross and mix (Fig. 4a). In contrast, the same type of projection performed on the SAS trials reveals an orderly relation within trial types (Fig. 4b). Namely, the position of a trial in the SAS extends throughout a single trial and systematically differs from trial to trial. Therefore, ALM population activity maintains a neural correlate of single trial identity beyond what is dictated by the task (since trials within the same trial-type are identical in terms of the task).

**Fig. 5:**
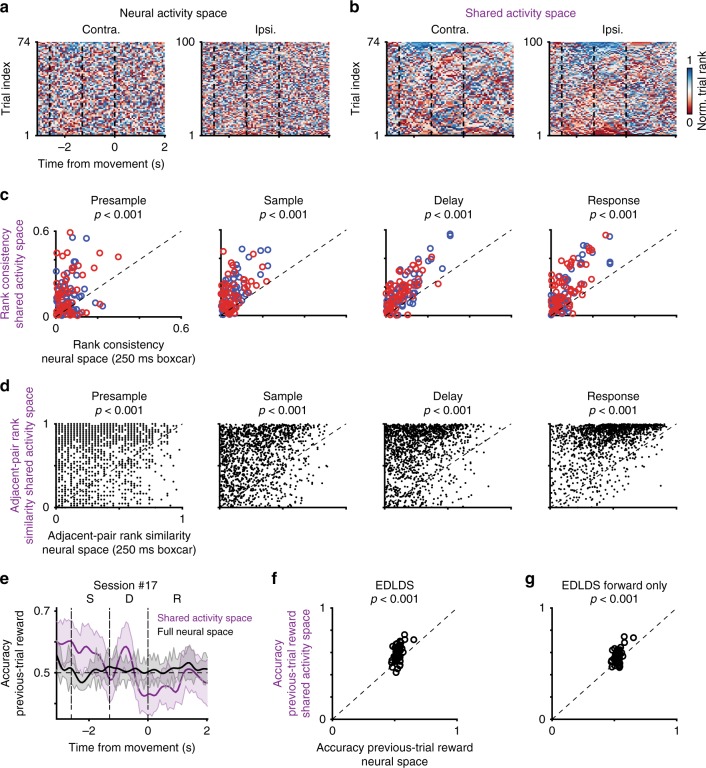
Long timescale structure of population activity revealed in shared activity space. **a** Heatmap of relative rank of population activity in full neural space for each trial type (74 contra, 100 ipsi trials). Rank at each time point was normalized to the maximum number of each trial type. **b** The same plot as **a** for activity in shared activity space. **c** Comparison of rank consistency across trials based on the averaged activity within each behavioral epoch in shared activity space (*y*-axis) versus full neural space (*x*-axis). Blue, contra.; red, ipsi. trials; each circle represents a single session. **d** Comparison of the rank-dynamics (change of within-epoch rank) consistency between two adjacent trials (1344 pairs across 55 sessions) in shared activity space (y-axis) versus full neural space (*x*-axis). **e** Decoding accuracy whether a previous trial was rewarded for decoders based on shared activity space in purple, for full neural space in black; shaded area, std. across trials. **f** Scatter of average decoding accuracy over pre-sample period for decoders based on full neural space (*x*-axis) or shared activity space (*y*-axis). Dash line, diagonal line. **g** The same convention as in **f** except comparing to shared activity space based on forward-only EDLDS fit (*y*-axis). All *p* < .001, sign-rank test

To better understand the neural correlates of single trial identity we tracked neural activity across time in single trials (Fig. 4) and considered their relationship across trials (Fig. 5) by analyzing the dynamics of the projections on decoders of trial type, since we found them highly predictive of behaviors^11^. We transformed the projection from its raw value to a relative rank across trials (either combining both trial types or separately per trial-type). For instance, if the projected values on trials 1–3 at a given time were 0.5, 1.3, and 0.2 the rank at that time would be 2,1,3, respectively. In FNS, separation between trial-type was consistent across time, but the rank within trial-type was not consistent (Fig. 4cd). In contrast, for decoders built from SAS we found single trial projections were both separated across trial types and maintained their ranks across time (Fig. 4ef). That is, a trial that ranked high at some time (high value of the projection on the decoder) tended to maintain a high rank during the rest of the trial. Even when averaging activity across an entire epoch to improve signal-to-noise, the single-trial rank correlations were significantly higher in SAS than in FNS across all epochs (*p* < .001, sign-rank test, Fig. 4g). This was not simply due to temporal smoothing in the SAS; decoders based on smoothed activity had weaker single-trial rank continuity (Supplementary Fig. 5c). Nor was this effect solely due to de-noising by pooling across neurons; single-trial rank consistency in the EDLDS-SAS was also superior to factor analysis, (Supplementary Fig. 5d), implying that interaction among latent modes is related to the continuity of dynamics. Comparing across models, the EDLDS model shows a stronger consistency of single-trial rank than most other models (Supplementary Fig. 6).

We were concerned the increased consistency in rank might stem from the non-causal nature of fitting latent dynamical system models, which infer the latent state at a given time based on all time points both in the past and future. We addressed this concern by multiple controls. First, running the EDLDS model in a strictly causal mode (i.e., using past time only to infer the current latent state, typically referred to as forward-pass), the rank remained significantly more consistent across time than in FNS (Fig. 4h, *p* < .001, sign-rank test). Second, if rank continuity was merely a property the model imposed on the data, it should be only weakly dependent on structure found in the data. In contrast, we found that shuffling neurons across trials within a trial type–taking the activity for each neuron and replacing it by the activity from the same neuron but a different trial of the same trial type–significantly reduced rank continuity (*p* < .001, sign-rank test, Supplementary Fig. 7). Thus, we conclude the persistence of the rank of a trial across epochs is an underlying property of the data that is revealed by the de-noising offered by the EDLDS model, not an artifact of the model.

Next, we asked whether longer timescale signals were discernible in the structure of population dynamics. We hypothesized that signals over multiple trials, tens of seconds, might be revealed by similarity in the activity rank in temporally adjacent trials (e.g., the next and previous trial). Visualizing the rank of each trial across time and trials, we found hints of such structure in FNS (Fig. 5a) and clear signs of rank similarity across multiple trials in the SAS (Fig. 5b). Once again, this cannot be an analytical artifact since the latent state in the EDLDS is estimated separately for each trial, restricting the non-causal nature of the model. To quantify these observed slow dynamics across trials, we computed the linear correlation between the temporal order of the trial (e.g., first trial, last trial) and the relative rank of its activity (within the same trial-type). Performing this analysis separately for each epoch, we found rank consistency in the SAS (*r* = .17 ± .02, 74 contra. trials; *r* = .20 ± .02, 100 ipsi. trials, mean ± sem) was significantly stronger than those in the FNS (Fig. 5c, p < .001, sign-rank test, for all epochs) or others (Supplementary Fig. 8a). Interestingly, simpler models (i.e., LDS, GPFA, EDLDS) better revealed long-time scale dynamics, perhaps because overfitting full-switching sLDS models to short timescale dynamics obscures longer time-scale correlations.

We next considered an additional form of long timescale structure: consistency in how relative rank changes within an epoch (contrast with the consistency in the average rank of a trial considered above). For example, relative rank starts low and increases when activity ramps up during a trial. The overall relative rank within an epoch would differ if for instance activity ramps up during an epoch in some trials while remaining flat in others. We refer to the similarity of within-epoch rank changes as rank-dynamics consistency. We quantified rank-dynamics consistency by correlating the relative rank within an epoch between two trials:

$$r_s\left( {i,j} \right) = \frac{{\left| {\left( {{\mathrm{rank}}_{i,n} - \left\langle {{\mathrm{rank}}_i} \right\rangle _n} \right)\left( {{\mathrm{rank}}_{j,n} - \left\langle {{\mathrm{rank}}_j} \right\rangle _n} \right)} \right|}}{{\sigma \left( {{\mathrm{rank}}_{i,n}} \right)\sigma \left( {{\mathrm{rank}}_{j,n}} \right)}}$$

where *r*_*s*_(*i*, *j*) is the rank correlation of trial *i* and *j* in rank space for epoch *n* in the same trial type; $\left\langle \cdot \right\rangle _n$ is an average over times in epoch *n*; and $\sigma ( \cdot )$ is the standard deviation. We compared this quantity on neighboring pairs (*j* = *i* + 1) of the same trial-type only, which occur infrequently in a randomized trial type design (*n* = 1344 pairs across 55 sessions). We found that rank-dynamics similarity was significantly stronger than the similarity found in the FNS (Fig. 5d; *p* < .001, sign-rank test, for all four epochs). Comparing across all other models, we found that the rank-dynamics similarity in the EDLDS was stronger than all models in nearly all behavioral epochs. Furthermore, all other models had at least one epoch that failed to show significant rank-dynamics similarity (Supplementary Fig. 8b).

We hypothesized longer timescale features of the dynamics may relate to recent behavioral history. Indeed, we found we could reliably predict the previous trial’s outcome^27^ (inter-trial intervals vary from 8–20 s) during the pre-sample epoch from the SAS, but not the FNS (Fig. 5e–f; other model fits, Supplementary Fig. 9a). This was similarly true for other behavioral variables in previous trials, such as choice^36^, early lick, and stimulus^37^ (Supplementary Fig. 9). The even longer timescale dynamics (tens of trials Fig. 5b) may relate to more difficult to track changes in behavioral states such as arousal or thirst.

These results reveal rich structure in ALM dynamics on the EDLDS latent space. Neural activity evolves in a manner such that an individual trial can be robustly tracked from sample epoch through delay epoch to response. Moreover, the dynamics of population activity change slowly over timescales of many seconds, with the dynamics seen in a particular trial more similar to the dynamics of a temporally-adjacent trial than trials at other times, presumably due to the influence of a slow-changing internal state. Finally, analysis in the SAS allowed explicit decoding of longer timescale behavior.

### Error trials

**Fig. 6:**
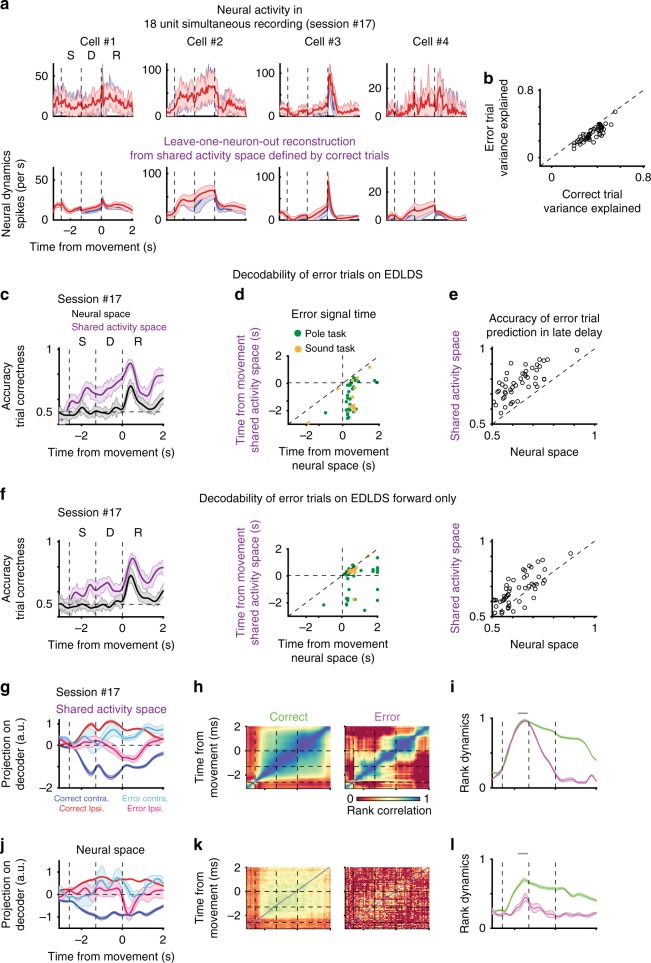
Error trials exhibit similar population dynamics to correct trials in the shared activity space. **a** Neuronal activity in error trials can be explained by a dynamical system model latent space estimated from correct trial data only. Top: observed neural activity in error trials (cells identical to those in Fig. 2b); bottom: prediction of the neural activity using leave-one-neuron-out methods based on a shared activity space fit using correct trials. Shaded area, std. across trials. **b** Fraction of variance explained by shared activity space model for error trials in different sessions (*y*-axis) scattered against fraction of variance explained by model on correct trials. **c** Decoding accuracy of trial-correctness for decoders based on shared activity space in purple, for full neural space in black; shaded area, std. across trials. **d** Scatter of time in which decoding accuracy reaches a selected threshold (.65) for decoders based on full neural space (*x*-axis) or shared activity space (*y*-axis). Green, pole task, delay starts at −1.3 s; Orange, sound task, delay starts at −2.0 s. **e** Scatter of decoding accuracy of trial correctness at late delay. Across sessions, decodability of trial correctness at late delay is high in SAS. Each circle corresponds to a session in **b**, **d** and **e**. **f** the same convention as those in **c–e**. except shared activity space is computed based on EDLDS forward-only pass (causal model). **g** Plot of neural dynamics in shared activity space projected on decoders of trial type for correct (blue, contra.; red, ipsi. trials) and error trials (cyan, contra.; magenta, ipsi. trials). Bold lines, averaged activity; shaded areas, sem. **h** Heatmaps of rank similarity in shared activity space across time averaged over correct (left) and error (right) trials. **i** Similarity of instantaneous rank to that in the late sample (−300 ms to 0 ms at the onset of delay, gray bar) in shared activity space averaged over correct (green) and error (magenta) trials. **j**–**l** the same convention as those in **g**–**i**, respectively, but for neural dynamics and its rank in full neural space

Given the unexpected degree of structure in the neuronal population dynamics, we hypothesized it might be relevant for understanding complex behavioral patterns such as error trials, where an animal makes an incorrect action. Error trial analysis can provide insight into the underlying computations^22^; they offer an opportunity to view a break in the typical, learned association between stimulus and action. Yet analyzing such trials is challenging both because they are rare and because the diverse causes for errors likely drive variable dynamics. Here, we compared the structure of dynamics in correct and error trials in the SAS. We hypothesized that the structure of dynamics for correct and error trials^38^ differs grossly, such that error trials would be marked by a failure of the LDS model derived from correct trials. Surprisingly, we found that SAS built from correct trials alone successfully predicted substantial variability in a LONO test during error trials (Fig. 6a; captured 52 ± 7% variance of that computed using PSTH, mean ± std.). This indicates that similar neural dynamics underlie the formation of correct and error trials, as in drift-diffusion models^39^. Additionally, although the fraction of explained variance was smaller in error trials (*p* < .001, sign-rank test; Fig. 6b), its value correlated strongly with the explained variance in correct trials. This indicates that the better the fit for the regular dynamics, the better the fit for the error-trial dynamics (*r*_s_ = .91, *p* < .001, sign-rank test), again in line with the notion that qualitatively similar dynamics underlie correct and error trials (Supplementary Fig. 10a, fit for other models).

If the structure of population dynamics is similar across both correct and error trials, what makes a trial an error trial? Knowing the behavioral contingency, one can spot error trials in motor or decision areas by neurons having a response more similar to the opposite behavioral contingency^22^. However, this schematic description of response flipping does not always capture the dynamics observed in many neurons and brain areas^38^. To discover errors that are driven by the neural representation being in a different state than correct trials (beyond the opposite sensory contingency), we performed a more challenging decoding analysis: decoding a trial’s correctness without access to its behavioral contingency (lick left or right). This decoding was far more effective in the SAS than in the FNS (Fig. 6c–e; *p* < .001, sign-rank test). In the SAS, accurate error decoding started early in the delay period (Fig. 6c; Session #17), whereas in the FNS there was no successful decoding of error until the delivery of reward. Across sessions, decoders decoded errors significantly earlier in the SAS (*p* < .001, sign-rank test; Fig. 6d). Furthermore, these results hold true when using the forward-only fit (*p* < .001, sign-rank test; Fig. 6f), implying they are not artifacts of the non-causal nature of the EDLDS model. Finally, the EDLDS model outperformed all other models in early predictions of error trials (Supplementary Fig. 10bc).

Comparing sessions with sufficient error trials for decoding, we observed the temporal profile of error decodability varied substantially from session to session (Supplementary Fig. 11). We explored this issue using two approaches. First, we compared the projection on decoders of trial type for correct and error trials; second, we inspected the rank-consistency we observed before. The average dynamics of error trials had diverse patterns, sometimes showing intermediate values between the trial conditions and sometimes showing a flip in the response to the other trial type (Supplementary Fig. 11b). The timing of the divergence in error trial dynamics to the direction opposite to correct trials was typically consistent with the previous decoder results (Fig. 6g). When analyzing rank consistency, we lacked sufficient numbers of error trials to analyze within-trial rank; therefore, we used a rank measure that incorporates both trial types. Specifically, we compared the rank of trials at each time point with a reference rank calculated around the transition between sample and delay (−300 ms to 0 ms relative to delay onset, qualitatively similar results were found for other time windows around this period). We found clear disruption in across-epoch rank-consistency in error trials (Fig. 6h), with rank dynamics starting to diverge between error and correct trials after the end of the sample epoch, consistent with the timing inferred from the other analyses (Fig. 6i). We cannot rule out that the distinct temporal profiles of error decodability are not the product of differential sampling of neural selectivity across sessions, but this cannot be directly controlled. Although we found no consistent patterns, there were sampling differences between sessions. For example, Session #17 has seven trial-type selective neurons (among 18 units) from sample to delay epochs, and error trial decodability held continuously across sample-delay epochs. In contrast, Session #13 (Supplementary Fig. 11) had one trial-type selective neuron in the sample epoch and four neurons (among 11 units) in the late delay, and error trial decodability broke down in the middle of delay. Performing the same analyses in the FNS (Fig. 6j–l), we found the divergence of error trials still agreed with the FNS decoder results, but the rank-consistency was too noisy to reliably compare to the timing of significant decoding (results for other sessions, Supplementary Fig. 11). In summary, the de-noising properties of the SAS allowed more fine-grained analysis of error related signals, with the inferred timing of hypothesized error generation consistent among multiple measures.

## Discussion

Neural dynamics are rich and heterogeneous, with large variability in responses both within a trial and across trials. Thus, describing the underlying computations and their relation to behavior is challenging. Here, we revealed stable, ordered internal representations that bridge these heterogeneities. We successfully applied latent dynamical system models to the epoch-dependent nature of the observed dynamics and showed these models can serve as proxies for internal states while accurately explaining behavior in both correct and error trials.

Our EDLDS model accurately predicted multiple features of behavior by judiciously pooling population activity across time and neurons into a lower dimensional representation, the shared activity space. Although the SAS has less information than the full neural space, it delivered superior performance compared to FNS in predicting multiple behavioral features. Here we discuss two explanations for this.

If the SAS captures most of the relevant information from the FNS with fewer parameters, SAS-trained decoders will generalize better than FNS-trained ones. Importantly, latent dynamical system models, such as EDLDS, capture information both across neurons and time, unlike static decoders. Attempting to capture all information without compression requires a large number of variables, e.g., a 4-s trial subdivided into 50-ms bins yields 80 temporal bins for the activity of a neuron; recording 20 neurons thus resulting in 1600 variables. The parameters of these variables are typically fit based on the data from a few hundred trials which can lead to poor model fitting. Regularization can alleviate some difficulties, but when a dataset’s correlation and noise structure are unknown, choosing effective, generally-applicable regularization methods is difficult^40^. In contrast, the EDLDS latent variables have lower dimensionality and their values at any time naturally incorporate past information, even when decoders only have access to data from a single time bin. This greatly reduces the number of parameters and improves generalization.

SAS performance may also benefit from imposing the assumption that population activity follows reliable dynamics, which can alleviate the effect of measurement noise. Consider a system for which the dynamics must a priori obey some rules. If our expectedly noisy observations of this system produce a trajectory inconsistent with known dynamics (e.g., a dropped ball observed to fly up), observational error must have occurred; thus, the true underlying state was not the one observed but rather another trajectory consistent with the known dynamics (e.g., gravity). Discounting this presumed erroneous observation is known as de-noising. For neural data, analyses that incorporate de-noising are likely to be more robust since spikes induce substantial measurement noise. Since the dynamics need to be learned from limited data, simpler models are preferred; yet if models of dynamics are too impoverished, imposing their inferred dynamics on the data will introduce bias instead of denoising. We hypothesized that adding event-driven switches to linear dynamical systems, generating the EDLDS model, would generate a rich and robust model of dynamics and thus allow effective de-noising even when estimating the dynamics from the data. Our results demonstrate the EDLDS model’s ability to assimilate and de-noise data, making it especially apt for performing single-trial population analysis^14–18,24–27,31^.

**Fig. 7:**
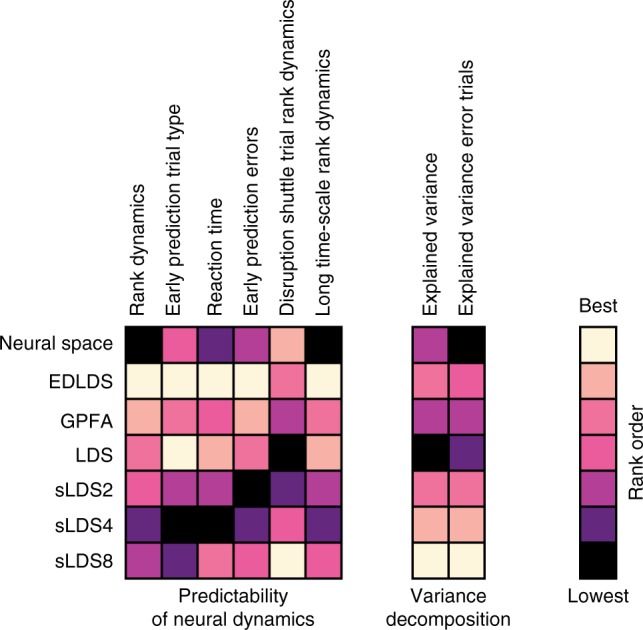
Summary of comparison across models. Results from Fig. 2 to Fig. 6 were compared across models and summarized into a pairwise comparison table. The comparison is divided into two categories according to the nature of the analyses. The first category emphasizes the predictability of behavioral and neural variability from different models. The analyses in this category includes: (1) rank dynamics: Fig. 4g, Supplementary Fig. 6; (2) early prediction of trial type: Fig. 3b, Supplementary Fig. 3a; (3) reaction time: Fig. 3d, Supplementary Fig. 3b; (4) early prediction of errors: Fig. 6d, Supplementary Fig. 10b; (5) disruption shuffle trial rank dynamics: Supplementary Fig. 7; (6) long time-scale rank dynamics: Fig. 5cd, Supplementary Fig. 8. The second category emphasizes the variance explained by each model. This includes the explained variance for correct trials (Fig. 2d) and error trials (Fig. 6b, Supplementary Fig. 10a)

Our results also illuminate how diverse population activity models capture neural activity and relate it to behavior, an important question given that most studies interpret neural dynamics through statistical models of differing complexity. A key measure of any model is its ability to fit the data, which can be readily quantified by predictions on held-out data. As expected, we found that more complex models perform better in explaining variance of held-out data, but these models did not explain behavioral variance better and revealed less underlying structure in dynamics (Fig. 7). Specifically, we observed many cases where the inferred switches in the latent dynamics of an sLDS model were not easily explained (Supplementary Fig. 2cd), perhaps because these are overfit, irrelevant switches that disrupted and obscured the structure of population dynamics. We also observed a stark contrast between how easily these models are to fit. Fitting LDS or our EDLDS models is conceptually and algorithmically simpler than sLDS models, and it is several orders of magnitude faster (in our hands, computation time for a typical set of recording sessions took weeks in sLDS versus a few hours in EDLDS). More generally, the difference between EDLDS and sLDS can be thought of as using supervised versus unsupervised inference of switches. In situations when a given behavioral event is likely to lead to a switch in dynamics, e.g., a change in behavioral epoch, one’s methodology should explicitly take this into account (e.g., EDLDS). The dataset we use in the paper will become freely available and has several interesting and non-trivial properties that may make it a useful benchmark or reference point for testing new models (Fig. 7).

Error trial analysis^22,23,34^ stands to greatly benefit from latent space approaches that allow more powerful single trial analysis. However, the small number of error trials renders fitting a dynamical system model of them unfeasible. If errors are caused by a strong change in the dimensionality of a representation^38^, adopting the same underlying dynamic model from correct trials would be inappropriate. Intuitively, at least some forms of error, e.g., lack of alertness, would also affect the underlying computations, again rendering them distinct from correct trials. To our surprise, the dynamical structure of population activity is unexpectedly similar in correct and error trials. Conceptually, this provides evidence against models that assume dynamics are distinct in error trials while bolstering other models in which error trial dynamics are more similar to correct trial dynamics, such as drift-diffusion models^39^ and some attractor models^41^. Practically, this means most latent dynamical system models can be fit with the number of correct trials to be reliably constrained for the small number of error trials. Importantly, we stress that comparing latent dynamics in correct and error trials goes beyond (and is complementary to) single-neuron error-trial analysis, and is only possible at the population level^42^. Future experiments that allow independent analysis of whether and when an animal is likely to make a mistake will be important to assess the advantages of finer-grained error-trial information revealed by latent space analysis and particularly in the non-trial-type-conditioned error analysis we performed.

In trial-based designs, one can compare how behavior differs across diverse trial conditions to the cognate trial-averaged population activity. Similarly, one can perform decoding analysis to regress neural activity against measured behavioral variables such as the instantaneous position or velocity of an animal. Such decoding analyses are robust for aspects of behavior that we directly impose or can reliably measure, such as trial condition (imposed) or position (measured). Yet an animal has additional internal states that affect behavior such as alertness etc. that can only be partially deduced from observation, e.g., measuring pupil diameter as a proxy for alertness. These states need not remain a black-box. Assuming they influence neural activity, they might be identified or inferred from population recordings^43^. Central hubs like decision-making areas (e.g., ALM) are key candidates for such analyses where long timescale dynamics might be revealed since they likely integrate many types of information and their activity is directly related to behaviors^11^.

Long timescale organization of activity is of particular interest both due to its temporal match with internal states that are thought to be long-lasting, and from the technical perspective of inferring structure in limited signal-to-noise sampling. In principle, such structure might be observed from single neurons or population activity (particularly when a behavior is correlated strongly with its previous trial^36,37,44^), but it may be obscured by the noise induced by spikes, especially in neurons with low firing rates. Here, we found strong evidence for such structure both across the seconds-long timescales expected from the nature of the task, i.e., transferring information from sample, delay to response, and across multiple trials, at hundred seconds. This organization can be faintly observed in the raw neural activity, but the denoising offered by EDLDS strongly and significantly unmasks this structure. In our study, sharp changes in neural dynamics occur in a coordinated fashion across trials, such that a candidate neural signature of a particular trial’s identity is maintained. Specifically, dynamics at distinct timescales do not interfere with each other; they also possibly reflect distinguishable computations: short timescale ones, task-related computations; long timescale ones, slow-varying internal brain states. This reveals an additional level of organization in neural activity and a potential leverage point for understanding long timescale internal state dynamics across brain areas and for understanding longer timescale phenomena such as learning, alertness, thirst, hunger or motivation.

## Methods

### Behavior

Mice were trained to perform a delayed version of a two-alternative forced-choice discrimination task^11^. Mice reported the position of a pole (anterior or posterior) or the frequency of a sound (low or high) by directional licking (lick-left, ipsi trial; or lick-right, contra trial) after a delay period (sample period: 1.3 s, delay period: 1.3 s, pole task; sample period: 1.15 s, delay period: 2.0 s, sound task). Inter-trial intervals were variable, ranging from 8–20 s from the previous trial’s go-cue. In addition, the next trial was not initiated until at least 4 s had passed after mice stopped licking. Mice were water restricted. Delivery of water began immediately following the detection of a correct lick. Reinforcement was not delayed and water was consumed during subsequent licking. Mice were trained to a criterion of at least 70% correct.

### Electrophysiological recordings

Electrophysiological recordings were performed on the left-hemisphere anterior lateral motor cortex (ALM) using 32-channel NeuroNexus silicon probes (*n* = 19 mice) or 64-channel Janelia silicon probes (*n* = 20 mice). Details of electrophysiology and spike sorting were described in^10,12,13^. Regardless some sessions were reported^10,12,13^, all sessions are compiled and released to [10.6084/m9.figshare.7372898]^45^ with codes (github.com/zqwei/TLDS_ALM_Data) that can recreate figures in the main text.

We excluded trials with early licking. Cells were previously classified as fast-spiking interneurons and pyramidal cells in^10,13^. Recording sessions were chosen based on two criteria: each session must have more than 5 units and the number of correct trials should be more than double the number of units (8 sessions in 32-channel recordings; 47 sessions in 64-channel ones). Summary is shown in Supplementary Table 1. Session #17 was used as the example session throughout the main text. This session contained 18 simultaneously recorded neurons with 14 pyramidal cells and 4 interneurons.

### Single neuron analysis

For all analyses, we binned neural activity using 67-ms discrete time window expect for when we explicitly compared to longer windows as a control in Fig. 4g, Supplementary Fig. 5c, where we used 250-ms bins in 10-ms steps. To compare the single trial variability in different neural spaces, we computed *std*. of neural dynamics within the same trial type; except for in Supplementary Fig. 1g-i; m-o, where sem. are presented.

To measure the dynamics of selectivity, we performed two-sample t tests with neural activity in each 67 ms discrete bins (Fig. 1b). We defined a neuron as monophasic if it had consistent polarity of selectivity (*p* < .05) for >335 ms (5 continuous bins), a neuron as multiphasic if it had a switch of selectivity (*p* < .05) with the periods of selectivity being at least 335 ms. The rest of the neurons were considered as nonselective neurons. For monophasic-selective neurons, we classified them into contra.- and ipsi.-preferring cells, according to the trial type for which they had higher activity (*p* < .05, two-sample t-test). Instead of correcting for multiple comparisons due to independent tests at each time bin we consider a neuron significant only if it had a significant value at 5 consecutive bins.

### Event-dependent linear dynamical system model

Our Event-Dependent Linear Dynamical System (EDLDS) model, is an extension of a linear latent space dynamical system model^30^. In such models, the full neural activity, $\left( {{\boldsymbol{r}}\left( t \right) \in {\boldsymbol{R}}^N} \right)$, is modeled as a projection up from a low dimensional neural-mode space, $\left( {{\boldsymbol{x}}\left( t \right) \in {\boldsymbol{R}}^M,\,M \, < \, N} \right)$ by a projection matrix, ***W***^**proj**^. The low dimensional modes evolve according to linear dynamics, ***W***^**mode**^, with Gaussian innovations (Eqs. 1 and 2). In our model the model parameters are fixed in time but are allowed to be different for each experimental epoch, *s*. This model is a compromise between models in which there is only one fixed latent model, which have dynamics that are incapable of capturing the rich dynamics we find in ALM and models that allow switching at any time point^18^, which suffer from difficulties in estimation due to the combinatorial number of possible hidden states of transitions over time. Under this model, the joint probability of the data and latent modes is:

3 $$P\left( {\left\{ {{\boldsymbol{x}}\left( t \right)} \right\},\left\{ {{\boldsymbol{y}}\left( t \right)} \right\}} \right) = P\left( {{\boldsymbol{x}}\left( 1 \right)} \right)\mathop {\prod }\limits_{t = 2}^T P\left( {{\boldsymbol{x}}\left( t \right)\left| {\boldsymbol{x}} \right.\left( {t - 1} \right)} \right)\mathop {\prod }\limits_{t = 1}^T P\left( {{\boldsymbol{r}}\left( t \right) - {\boldsymbol{r}}_0\left| {\boldsymbol{x}} \right.\left( t \right)} \right),$$

where ${\boldsymbol{r}}(t) - {\boldsymbol{r}}_0\left| {\boldsymbol{x}} \right.\left( t \right) \equiv {\bar{\boldsymbol r}}\left( t \right)\sim N\left( {{\boldsymbol{W}}^{{\boldsymbol{proj}}}(s){\boldsymbol{x}}\left( t \right),\,{\boldsymbol{Q}}_{ext}\left( s \right)} \right)$, ${\boldsymbol{x}}(t)\left| {\boldsymbol{x}} \right.\left( {t - 1} \right)\sim N\left( {{\boldsymbol{W}}^{{\boldsymbol{mode}}}\left( s \right){\boldsymbol{x}}\left( {t - 1} \right),\,{\boldsymbol{Q}}_{int}(s)} \right)$, and the initial state of the neural mode ${\boldsymbol{x}}\left( 1 \right)\sim N\left( {{\boldsymbol{x}}_0,\,{\boldsymbol{Q}}_0} \right)$.

The parameter set $\Theta$ included the amplitudes of external independent inputs ${\boldsymbol{Q}}_{ext}(s) = \left\{ {\sigma _{ext}^2\left( n \right)} \right\}$ onto each neuron ($1 \le n \le N$; *N*, the number of neurons in simultaneous recordings), the amplitude of internal inputs ${\boldsymbol{Q}}_{int}(s) = \left\{ {\sigma _{int}^2\left( m \right)} \right\}$ onto each neural mode ($1 \le m \le M$; *M* < *N*, the latent dimension), the latent connectivity, ${\boldsymbol{W}}^{{\boldsymbol{mode}}}\left( s \right) \in {\boldsymbol{R}}^{M \times M}$, and the projection, ${\boldsymbol{W}}^{{\boldsymbol{proj}}}\left( s \right) \in {\boldsymbol{R}}^{N \times M}$ for each epoch (i.e., pre-sample, sample, delay, and response), and those associated with the initial state of the neural mode ***x***_0_, ***Q***_0_ in trials.

The estimation of the EDLDS followed similar Expectation-Maximization steps as that in ref. ^14^. The expectation step was identical and the maximization step was:

4 $$\begin{array}{ccccc}\\ & {\boldsymbol{W}}^{{\boldsymbol{proj}}\, \ast }\left( s \right) = \left( {\mathop {\sum }\limits_{t = t\left( {s - 1} \right)}^{t\left( s \right)} {\bar{\boldsymbol r}}_t{\boldsymbol{x}}_t^T} \right)\left( {\mathop {\sum }\limits_{t = t\left( {s - 1} \right)}^{t\left( s \right)} {\boldsymbol{x}}_t{\boldsymbol{x}}_t^T\left| {\left\{ {{\bar{\boldsymbol r}}_t} \right\}_{1 \le t \le T}} \right.} \right)^{ - 1},\\ \\ & {\boldsymbol{Q}}_{ext}^ \ast \left( s \right) = \frac{1}{{t\left( s \right) + 1 - t(s - 1)}}\mathop {\sum }\limits_{t = t\left( {s - 1} \right)}^{t\left( s \right)} \left( {{\bar{\boldsymbol r}}_t - {\boldsymbol{W}}^{{\boldsymbol{proj}}\, \ast }\left( s \right){\boldsymbol{x}}_t} \right){\bar{\boldsymbol r}}_t^T,\\ \\ & {\boldsymbol{W}}^{{\boldsymbol{mode}}\, \ast }\left( s \right) = \left( {\mathop {\sum }\limits_{t = t\left( {s - 1} \right) + 1}^{t\left( s \right)} {\boldsymbol{x}}_t{\boldsymbol{x}}_{t - 1}^T\left| {\left\{ {{\bar{\boldsymbol r}}_t} \right\}_{1 \le t \le T}} \right.} \right)\\ \\ & \left( {\mathop {\sum }\limits_{t = t\left( {s - 1} \right) + 1}^{t\left( s \right)} {\boldsymbol{x}}_{t - 1}{\boldsymbol{x}}_{t - 1}^T\left| {\left\{ {{\bar{\boldsymbol r}}_t} \right\}_{1 \le t \le T}} \right.} \right)^{ - 1},\\ \\ & {\boldsymbol{Q}}_{int}^ \ast \left( s \right) = \frac{1}{{t\left( s \right) - t\left( {s - 1} \right)}}\\ \\ & \left( {\mathop {\sum }\limits_{t = t\left( {s - 1} \right) + 1}^{t\left( s \right)} {\boldsymbol{x}}_t{\boldsymbol{x}}_t^T\left| {\left\{ {{\bar{\boldsymbol r}}_t} \right\}_{1 \le t \le T}} \right. - {\boldsymbol{W}}^{{\boldsymbol{mode}}\, \ast }\left( s \right)\mathop {\sum }\limits_{t = t\left( {s - 1} \right) + 1}^{t\left( s \right)} {\boldsymbol{x}}_{t - 1}{\boldsymbol{x}}_{t - 1}^T\left| {\left\{ {{\bar{\boldsymbol r}}_t} \right\}_{1 \le t \le T}} \right.} \right).\\ \end{array}$$

where *t*(*s*) presents the last time point of epoch *s* and *t*(0) = 1. Here we constrained the estimation of input matrices ${\boldsymbol{Q}}_{ext}(s)$ and ${\boldsymbol{Q}}_{int}(s)$ to be diagonal after each iteration of maximization. Further, we computed dynamics of the neural mode in the shared activity space as ${\boldsymbol{x}}_t\left| {\left\{ {{\bar{\boldsymbol r}}_t} \right\}_{1 \le t \le T}} \right.$ from the expectation step. Model estimation was based only on correct trials.

### Leave-one-neuron-out estimation

To determine the performance of model fits, we computed the variance explained using a leave-one-out procedure in cross-validation^15^. After fitting the model on training data, we take test data, remove the activity of the *i-*th neuron from the data, hence leave-one-neuron-out (LONO), and estimate the values of the shared activity space modes: ${\boldsymbol{x}}_t\left| {\left\{ {{\bar{\boldsymbol r}}_t^{ - i}} \right\}_{1 \le t \le T}} \right. \cong {\boldsymbol{x}}_t\left| {\left\{ {{\bar{\boldsymbol r}}_t} \right\}_{1 \le t \le T}} \right..$ We then compute the expected activity of the *i*th neuron as $\hat r_t^i = {\boldsymbol{W}}^{{\boldsymbol{proj}}\,i}(s){\boldsymbol{x}}_t\left| {\left\{ {{\bar{\boldsymbol r}}_t^{ - i}} \right\}_{1 \le t \le T}} \right. + r_0^i$, where ${\boldsymbol{W}}^{{\boldsymbol{proj}}\,i}$ is the *i*th row of the projection matrix ***W***^***proj***^. The explained variance,

5 $$R^2 = 1 - \left\langle {\frac{{\left\langle {\left| {\left| {{\boldsymbol{r}}_t^i - {\hat{\boldsymbol r}}_t^{\boldsymbol{i}}} \right|} \right|_2^2} \right\rangle _t}}{{\left\langle {\left| {\left| {{\boldsymbol{r}}_t^i - {\boldsymbol{r}}_0} \right|} \right|_2^2} \right\rangle _t}}} \right\rangle _i$$

measured the goodness-of-fit (where $\left\| \cdot \right\|_2$, is the *L*^2^ norm of the vector; $\left\langle \cdot \right\rangle _t$, is an average over time and trials; $\left\langle \cdot \right\rangle _i$ is an average over neurons).

To select the dimensionality of the shared activity space, M*, we performed LONO estimation of TLDS fit using 10-fold cross-validation for each possible dimension, $1 \le M \le N - 2$ (Fig. 2c). The amount of the variance explained as a function of the dimensionality of the shared activity space first increased (corresponding to an under-fitting region) then saturated or even decreased (an over-fitting region). To avoid overfitting we picked the minimal dimension that reached a criterion of 90% of the maximum amount of variance explained. As a control we also compared results to the amount of variance explained in a model where either ***W***^***mode***^ or ***W***^***proj***^ was constant matrices (Fig. 2c).

As a comparison, we computed explained variance of a reference model, the mean-activity model, where we assume that neural activity follows a random fluctuation around its mean in each trial. Therefore, for each neuron, $\hat r_t^i\left( s \right) = \left\langle {r_t^i\left( s \right)} \right\rangle _i$, and index s stands for the trial type: correct contra, correct ipsi, error contra, or error ipsi trials. Computation of explained variance then follows Equation 5.

### Decoding analysis

To determine trial-type decoding we applied linear discriminant analysis (LDA) on neural dynamics grouped into 67-ms non-overlapped bins. The LDA decoder, ${\boldsymbol{l}}_t$, was computed separately for each time bin, t, using correct response trials only. The neural dynamics projected onto the decoder was then computed as $s_t = {\boldsymbol{l}}_t^T{\boldsymbol{r}}_t$, where ***r***_*t*_ is the vector of neural activity in time bin t. To avoid the ambiguity of sign in the decoder we assigned a sign to the decoder so that *s*_*t*_ < 0 in contra and *s*_*t*_ > 0 in ipsi trials. The same procedure was applied for decoders trained on the shared activity space. The correlation of projected activity across time, *t*, and, *t*′, was performed using Spearmen’s rank correlation, $r_s\left( {t,t^\prime } \right)$ and that across epochs, *s*, and, *s*′, were as $\left\langle {r_s\left( {t,t^\prime } \right)} \right\rangle _{t \in s;t^\prime \in s^\prime }$ (Fig. 4g, h, Supplementary Fig. 6). Epoch coding decoders were based on the neural dynamics averaged within each behavioral epoch; trial-coding direction is based on the neural dynamics averaged from sample to response epochs.

To achieve robust estimation given the large number of neurons, we applied a sparse version of LDA^35^, with normalization that $\left\| {{\boldsymbol{l}}_t} \right\|_2 = 1$ (where $\left\| \cdot \right\|_2$, the *L*^2^ norm of the vector) for each time point t in a task.

$${\boldsymbol{l}}_t = \arg \,\mathop {{\min }}\limits_{\boldsymbol{l}} - \frac{{\left( {{\boldsymbol{l}}^T\left( {{\boldsymbol{r}}_t^{contra} - {\boldsymbol{r}}_t^{ipsi}} \right)} \right)^2}}{{{\boldsymbol{l}}^T\Sigma _r{\boldsymbol{l}}}} + \Delta \left| {\boldsymbol{l}} \right|_1$$

Two parameters were used to regularize sparsity of LDA coefficients, *γ* (*l*_2_-regularizer of covariance matrix of sample data $\Sigma _r = \left( {1 - \gamma } \right)\Sigma + \gamma \Sigma$; $\gamma \in \left[ {0,1} \right]$; $\Sigma = \left( {{\boldsymbol{r}} - \left\langle {\boldsymbol{r}} \right\rangle } \right)\left( {{\boldsymbol{r}} - \left\langle {\boldsymbol{r}} \right\rangle } \right)^T$) and Δ (*l*_1_-regularizer of covariance matrix of sample data $\Delta \left| {\boldsymbol{l}} \right|_1$). The optimization of these two parameters follows the standard Matlab procedure (Supplementary Fig. 4) using cross validation (all trials were divided into training and testing sets), and the set of parameters minimizing the validation error were used in regularizing ***l***_*t*_ in final fit using all trials. In this case, the LDA coefficient should be close to zero if a neuron fired at a low rate or contributed little to coding trial type. We measured the similarity of the coding directions across time, *t*, and, *t*′, as ${\boldsymbol{l}}_t^T{\boldsymbol{l}}_{t^\prime }$. We did not observe any ${\boldsymbol{l}}_t^T{\boldsymbol{l}}_{t^\prime } < \, 0$.

### Decodability

The performance of trial-type decodability was computed based on the decoder projection in a 150-ms time bin at 300 ms before the onset of response using 10-fold cross-validation (Fig. 3ab; error bar, std.). We computed performance of trial-correctness decodability using two nonlinear decoders (Fig. 6c, f; Supplementary Fig. 11), one based on a 2nd order polynomial-kernel support vector machine (SVM; kernel function, $G\left( {r_t^i,r_t^j} \right) = \left( {1 + \left\langle {r_t^ir_t^j} \right\rangle } \right)^2$; *i*, *j*, neural index; *r*, neural activity; $\left\langle \cdot \right\rangle$, average over trials), the other based on quadratic discriminant analysis (QDA), using instantaneous neural dynamics in discrete 67-ms time bins, following 10-fold cross-validation (shaded area, std.). The correctness signal onset time (Fig. 4e) was computed as the first time when performance of trial-correctness decodability was continuously >.65 for at least one behavioral epoch. We then compared the single-trial decodability in FNS versus that in SAS using paired sign-rank test across trials.

### Reaction time correlations

We correlated decoder projection scores with reaction time to the first lick using Spearmen’s rank correlation. The decoder projection score was estimated by averaging over a 150-ms time bin at 300 ms before the onset of response (Fig. 3cd; Supplementary Fig. 3b).

## Supplementary information

Supplementary Information

Description of Additional Supplementary Files

Supplementary Table 1

## Data Availability

Code for this study is publicly available on Github: [https://github.com/zqwei/TLDS_ALM_Data]. Code for Event-dependent Linear Dynamical Systems can be separately got at [https://github.com/zqwei/Epoch-Dependent-LDS-Fit] [10.5281/zenodo.1403158]. Precompiled simultaneous recording sessions can be obtained at^45^ 10.6084/m9.figshare.7372898.
