# Supplementary material for: An orderly single-trial organization of population dynamics in premotor cortex predicts behavioral variability
Source: Nat Commun. 2019 Jan 15;10:216. doi: 10.1038/s41467-018-08141-6 (PMC6333792; doi:10.1038/s41467-018-08141-6)

# Supplementary Information

**An orderly single-trial organization of population dynamics in premotor cortex predicts behavioral variability**

**Wei et al.**

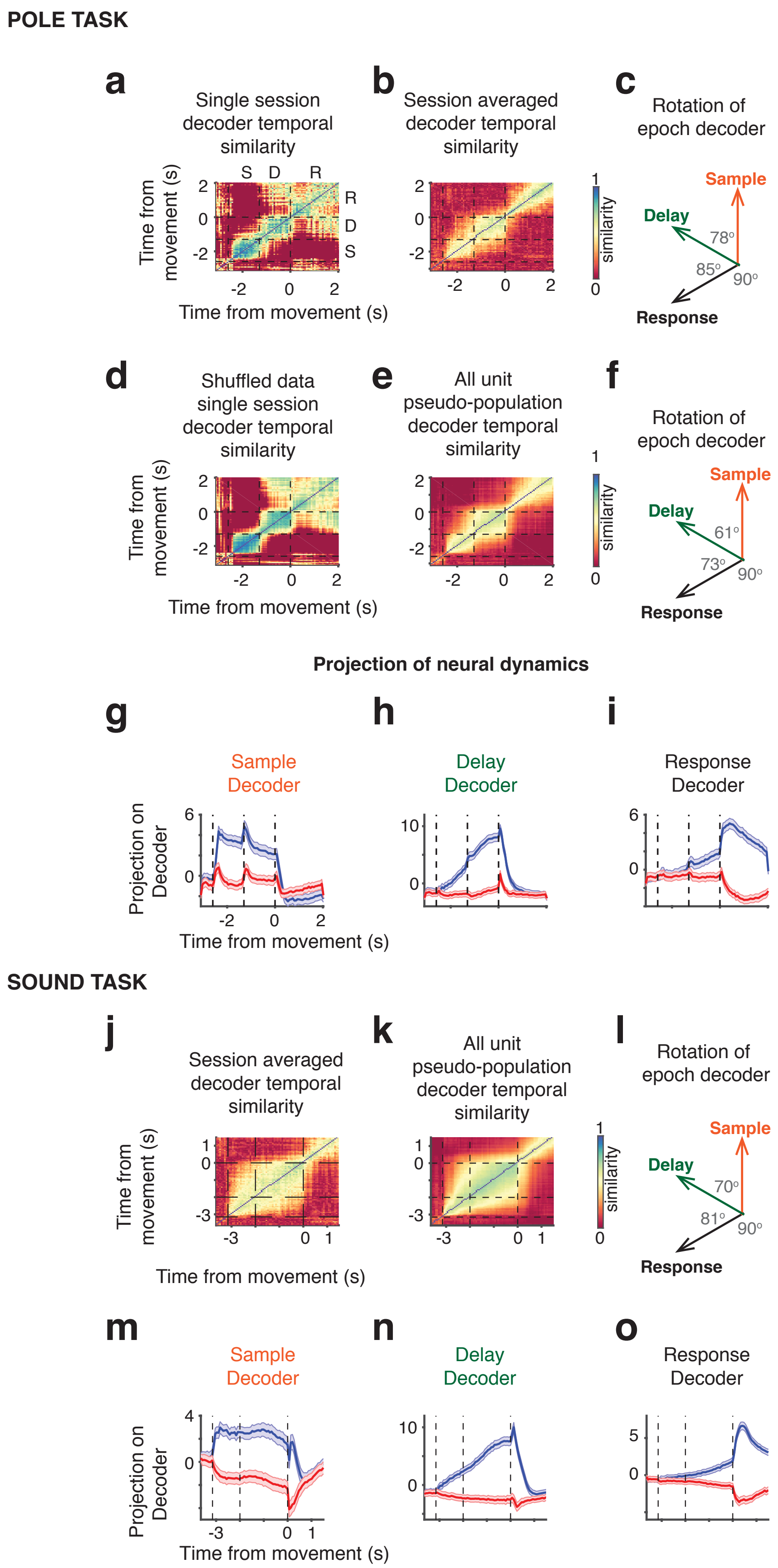

POLE TASK
a
Single session decoder temporal similarity
S D R
R D S
Time from movement (s)
b
Session averaged decoder temporal similarity
similarity
c
Rotation of epoch decoder
Sample
Delay
Response
78°
85°
90°
d
Shuffled data single session decoder temporal similarity
Time from movement (s)
e
All unit pseudo-population decoder temporal similarity
similarity
f
Rotation of epoch decoder
Sample
Delay
Response
61°
73°
90°
Projection of neural dynamics
g
Sample Decoder
Projection on Decoder
Time from movement (s)
h
Delay Decoder
i
Response Decoder
SOUND TASK
j
Session averaged decoder temporal similarity
Time from movement (s)
k
All unit pseudo-population decoder temporal similarity
similarity
l
Rotation of epoch decoder
Sample
Delay
Response
70°
81°
90°
m
Sample Decoder
Projection on Decoder
Time from movement (s)
n
Delay Decoder
o
Response Decoder

**Supplementary Figure 1. (Supporting figure for Figure 1) Rotation of coding directions and population dynamics in full neural space**

**a-f.** Rotation of coding directions in full neural space. **a-c**. Similarity of instantaneous trial-type decoders across time. Color bar on right indicates correlation between decoders at different times. Coding direction is similar within epoch but different from sample-delay epoch to response, indicating the discontinuous dynamics of selectivity at population level. **a.** Single session analysis (n = 18 neurons; Session #17). **b.** Same analysis averaged across sessions (n = 40 sessions; pole task). **c**. Schematic of the rotation of decoders across epochs and value of angles between epoch-averaged decoders. **d.** Temporal correlation between instantaneous decoders across time similarity plot, same session as **a**, but with shuffled trials. **e.** All unit pseudo-population temporal correlation between instantaneous decoders (n = 1,743 units, randomly shuffled pseudo-combined trials). **f.** Schematic of the rotation of decoders across epochs and value of angles between epoch-averaged decoders for the all-unit pseudo population. **g-i**. Projection of the pseudo-population neural activity on trial type decoders based on activity averaged across each behavioral epoch: sample (**g**), delay (**h**) and response (**i**); blue, contra.; red, ipsi. trials; solid line, average activity along the projection; shaded area, sem. **j-o.** The same convention as that in **a-i**, except for sound task.

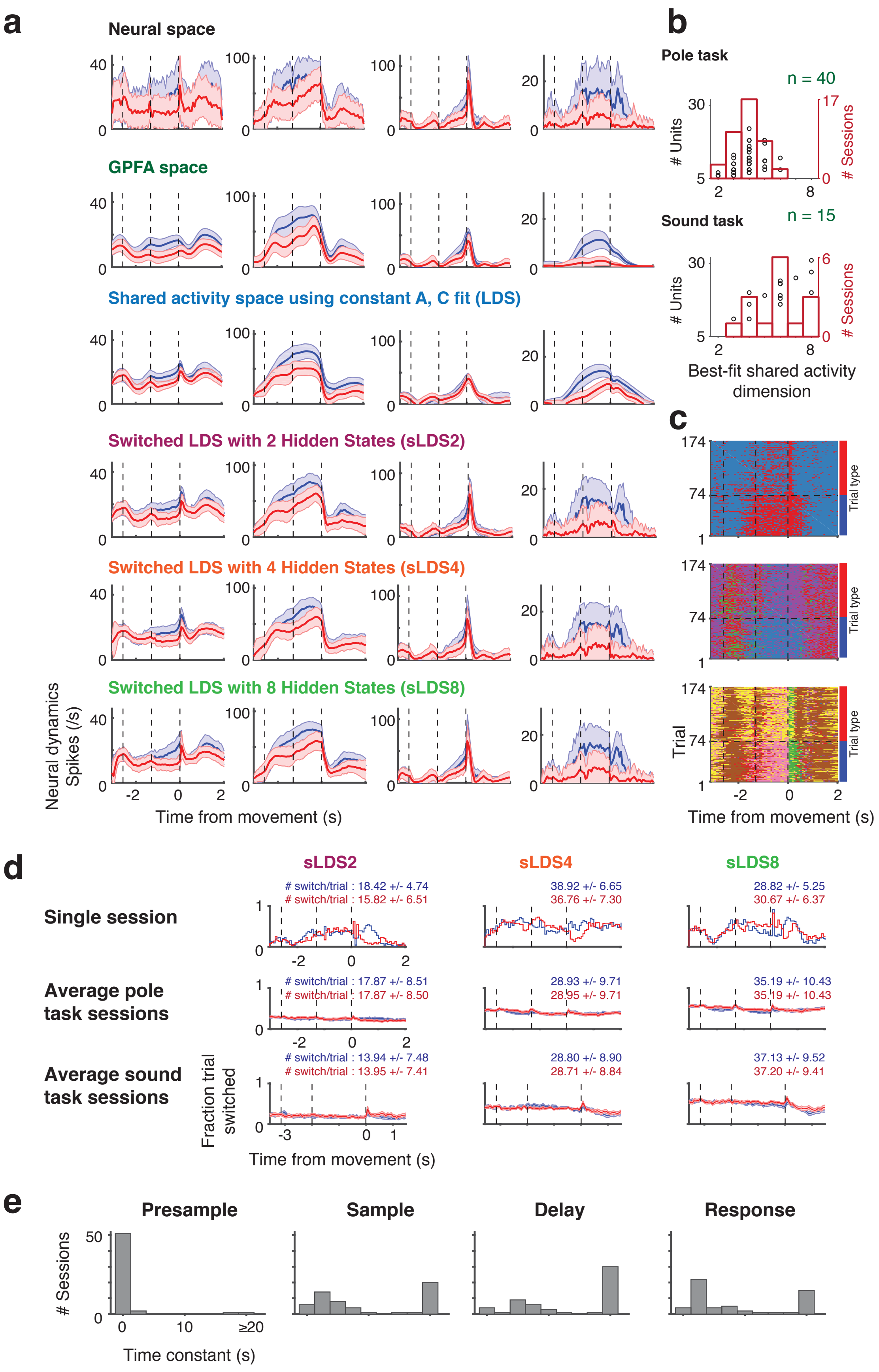

a
Neural space
GPFA space
Shared activity space using constant A, C fit (LDS)
Switched LDS with 2 Hidden States (sLDS2)
Switched LDS with 4 Hidden States (sLDS4)
Switched LDS with 8 Hidden States (sLDS8)
Neural dynamics
Spikes (/s)
Time from movement (s)
b
Pole task
n = 40
# Units
# Sessions
Sound task
n = 15
Best-fit shared activity dimension
c
Trial type
Trial
Time from movement (s)
d
sLDS2
sLDS4
sLDS8
Single session
# switch/trial : 18.42 +/- 4.74
# switch/trial : 15.82 +/- 6.51
38.92 +/- 6.65
36.76 +/- 7.30
28.82 +/- 5.25
30.67 +/- 6.37
Average pole task sessions
# switch/trial : 17.87 +/- 8.51
# switch/trial : 17.87 +/- 8.50
28.93 +/- 9.71
28.95 +/- 9.71
35.19 +/- 10.43
35.19 +/- 10.43
Average sound task sessions
# switch/trial : 13.94 +/- 7.48
# switch/trial : 13.95 +/- 7.41
28.80 +/- 8.90
28.71 +/- 8.84
37.13 +/- 9.52
37.20 +/- 9.41
Fraction trial switched
Time from movement (s)
e
Presample
Sample
Delay
Response
# Sessions
Time constant (s)

**Supplementary Figure 2. (Supporting figure for Figure 2) Neural activity fits on other models**

**a.** Fit of the same neuronal activity in **Figure 2b** using other models, following the same convention as that in **Figure 2b**. **b.** Variance saturating dimension (VSD) of latent space model for all sessions using EDLDS fit. Plot shows each session as a black circle; x-value, VSD for that session, y-value, number of units. Red bars show number of sessions with a given VSD. Top, pole task; bottom, sound task. **c.** Fit of hidden states in different sLDS models. Top, sLDS2 (2 hidden states); middle, sLDS4 (4 hidden states); bottom, sLDS8 (8 hidden states). Each color presents a unique state in heatmaps and fit allows the switch at any time during a trial. The average number of switches in each trial type were reported in **d**. **d**. Fraction of switch happens uniformly in time. **e**. Distributions of time constants of fits in EDLDS model for different behavioral epochs (tailed at 20 s). The time constants is computed based largest of eigenvalue of $\boldsymbol{W}^{\boldsymbol{mode}}$ where $\tau = \frac{\Delta T}{1-\lambda_{max}(\boldsymbol{W}^{\boldsymbol{mode}})}$; $\lambda_{max}(\boldsymbol{W}^{\boldsymbol{mode}})$ for each epoch in a session is reported in **Supplementary Table 1**; $\Delta T = 67$ ms is the time bin in our fits; for $\boldsymbol{W}^{\boldsymbol{mode}} > 1$, we consider its $\tau > 20$ s.

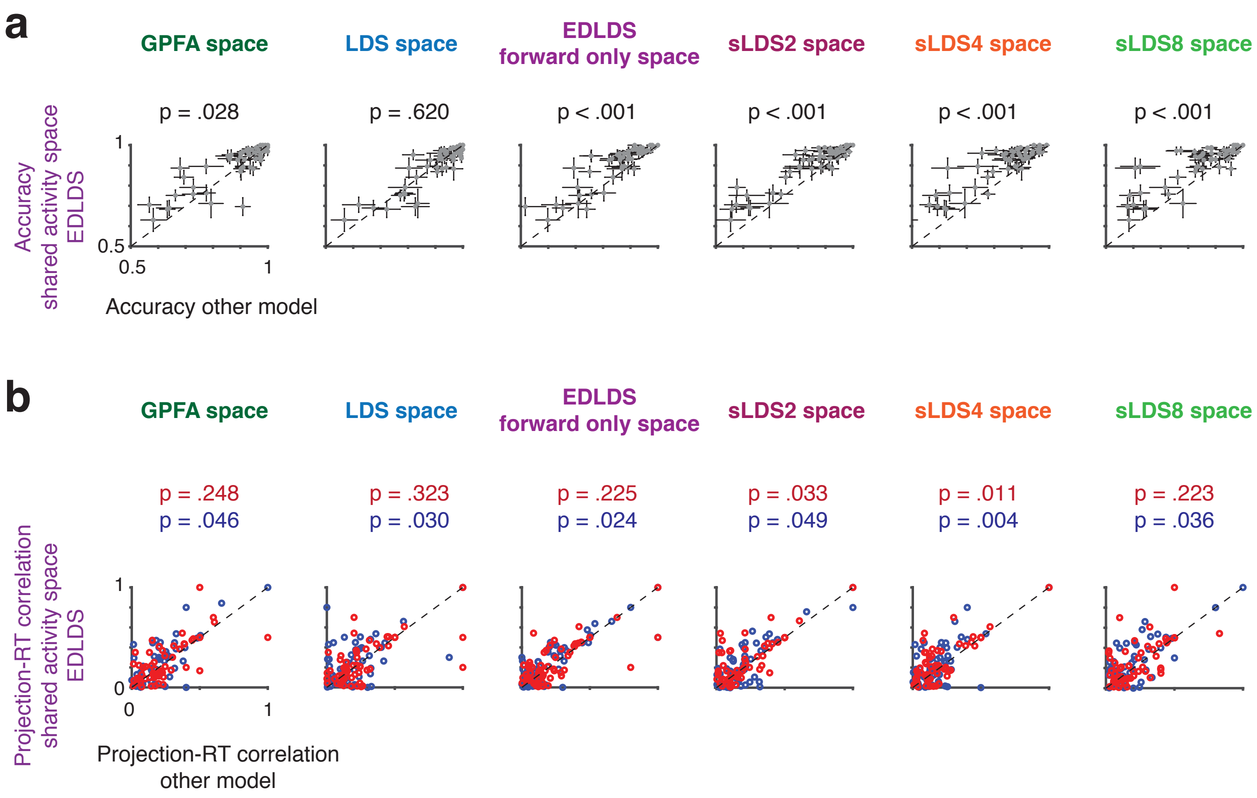


**Supplementary Figure 3. (Supporting figure for Figure 3)**

**a.** Following the same convention as that in **Figure 3b** except for other models. **b**. Following the same convention as that in **Figure 3d** except for other models. Both p-value is reported using sign-rank test.

**a**

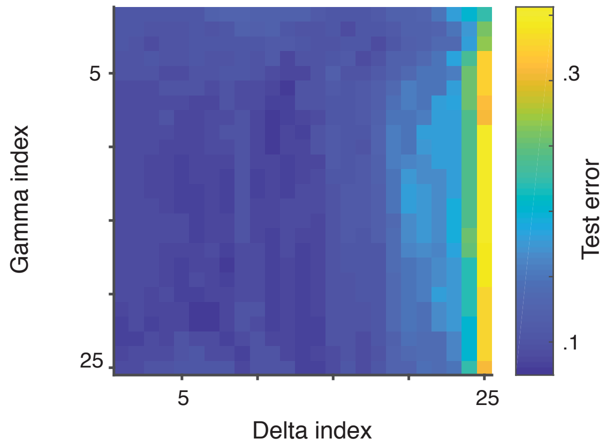


**b**

```
Matlab Codes:
Mdl = fitcdiscr(neuralActivity, trialType, 'discrimType', 'Linear');
[err, gamma, delta, numpred] = cvshrink(Mdl, 'NumGamma',24, 'NumDelta', 24)
# plot above
figure, imagesc(err)

# find optimal gamma and delta using min-min rule (Guo, Hastie, Tibshirani, 2007)
minerr = min(min(err))
[p,q] = find(err < minerr + 1e-4); % Subscripts of err producing minimal error
idx = sub2ind(size(delta),p,q); % Convert from subscripts to linear indices
optimal_gamma = gamma(p)
optimal_delta = delta(idx)
```

**Supplementary Figure 4. (Supporting figure for Methods) Regularization procedure on decoders**

**a.** Validation error on Gamma-Delta space (Methods) **b.** Matlab code for searching the optimal pair of Gamma-Delta parameters according to the validation errors.

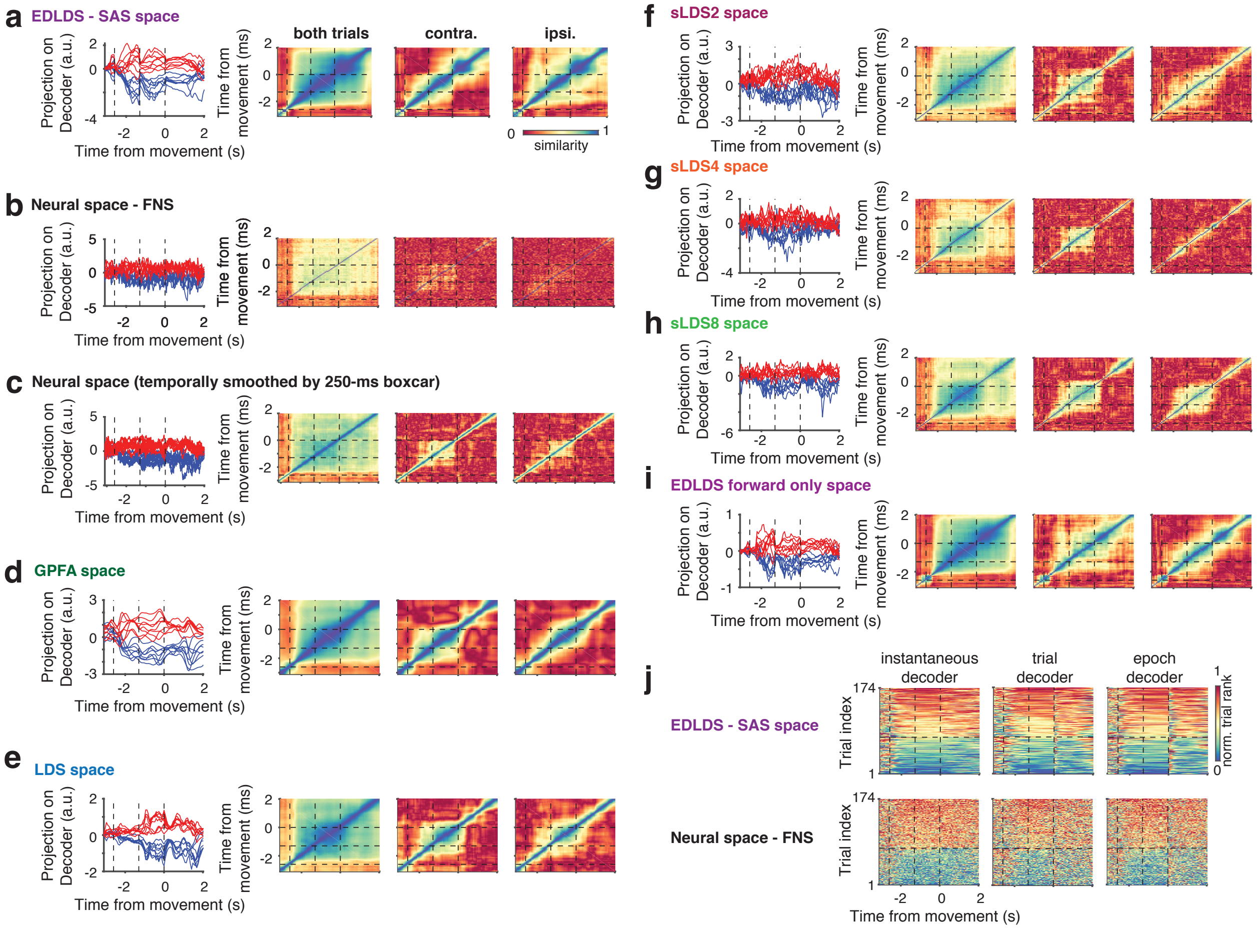


**Supplementary Figure 5. (Supporting figure for Figure 4c-f) Rank correlation of trials in shared activity space**

**a. left,** Traces of single trial projections for the two trial types onto trial type decoders based on the EDLDS shared activity space; **right**, Rank correlation between the neural projections onto the instantaneous coding direction at different time points, across both trial types (left), across only contra. trials (middle), and across only ipsi. trials (right). **b-i**. the same conventions as **a.** except those based on the full neural space, temporally smoothed full neural space (250 ms boxcared), Gaussian process factor analysis (GPFA) shared activity space, time-invariant linear dynamical systems (LDS) shared activity space, switched linear dynamical system with 2 hidden states (sLDS2), switched linear dynamical system with 4 hidden states (sLDS4), and switched linear dynamical system with 8 hidden states (sLDS8), respectively. **j.** Rank of all trials across time. Data sorted according to average rank in sample-delay epochs. Each plot uses projections on a different decoder: instantaneous decoders (left), decoder based on average activity from sample to response epoch (middle) and a decoder that changes in each epoch and uses the average activity in that epoch (right). Top, EDLDS shared activity space; bottom, full neural space.

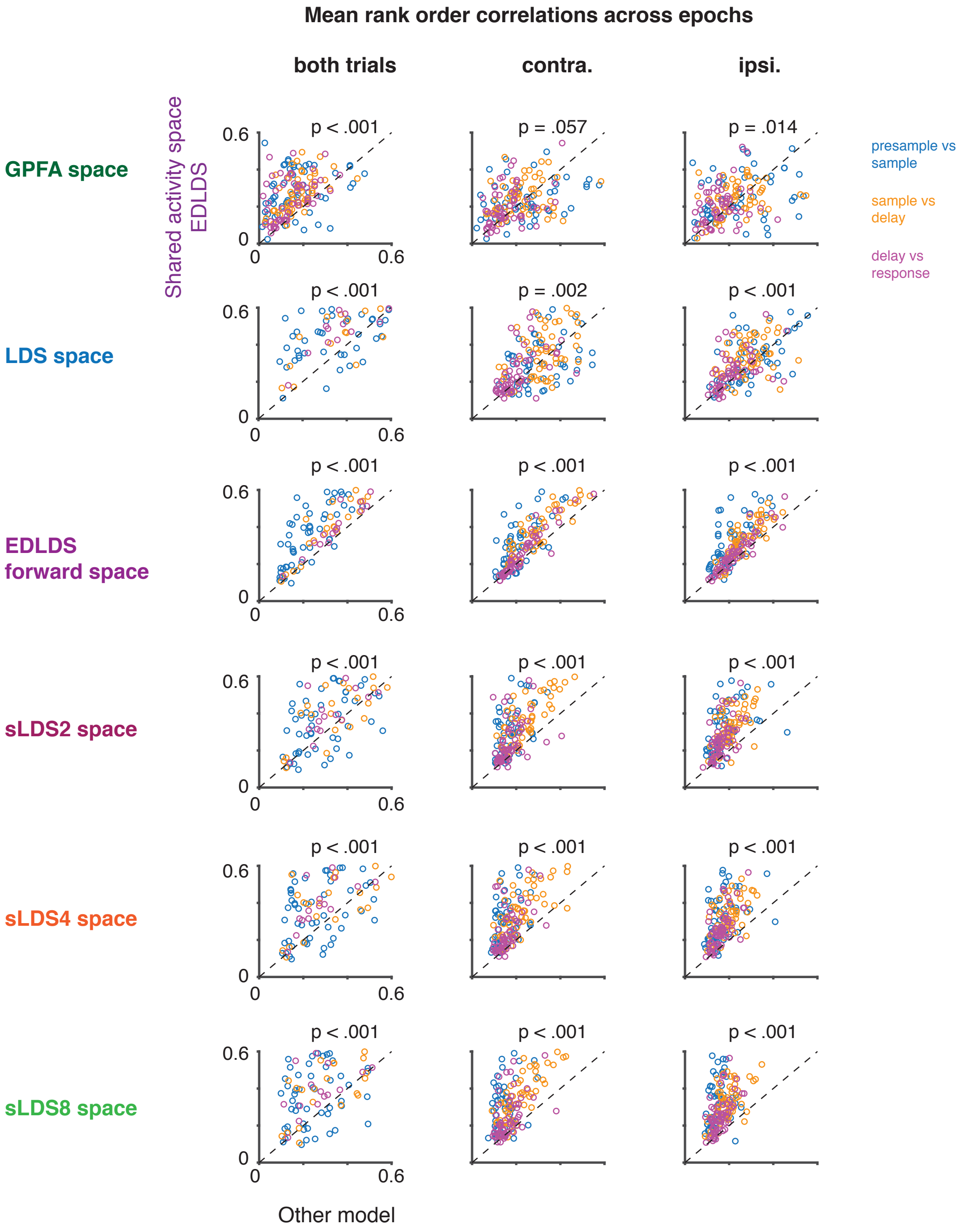


**Supplementary Figure 6. (Supporting figure for Figure 4g)**

Following the same convention as that in **Figure 4g** except for other models. P-value is reported using sign-rank test.

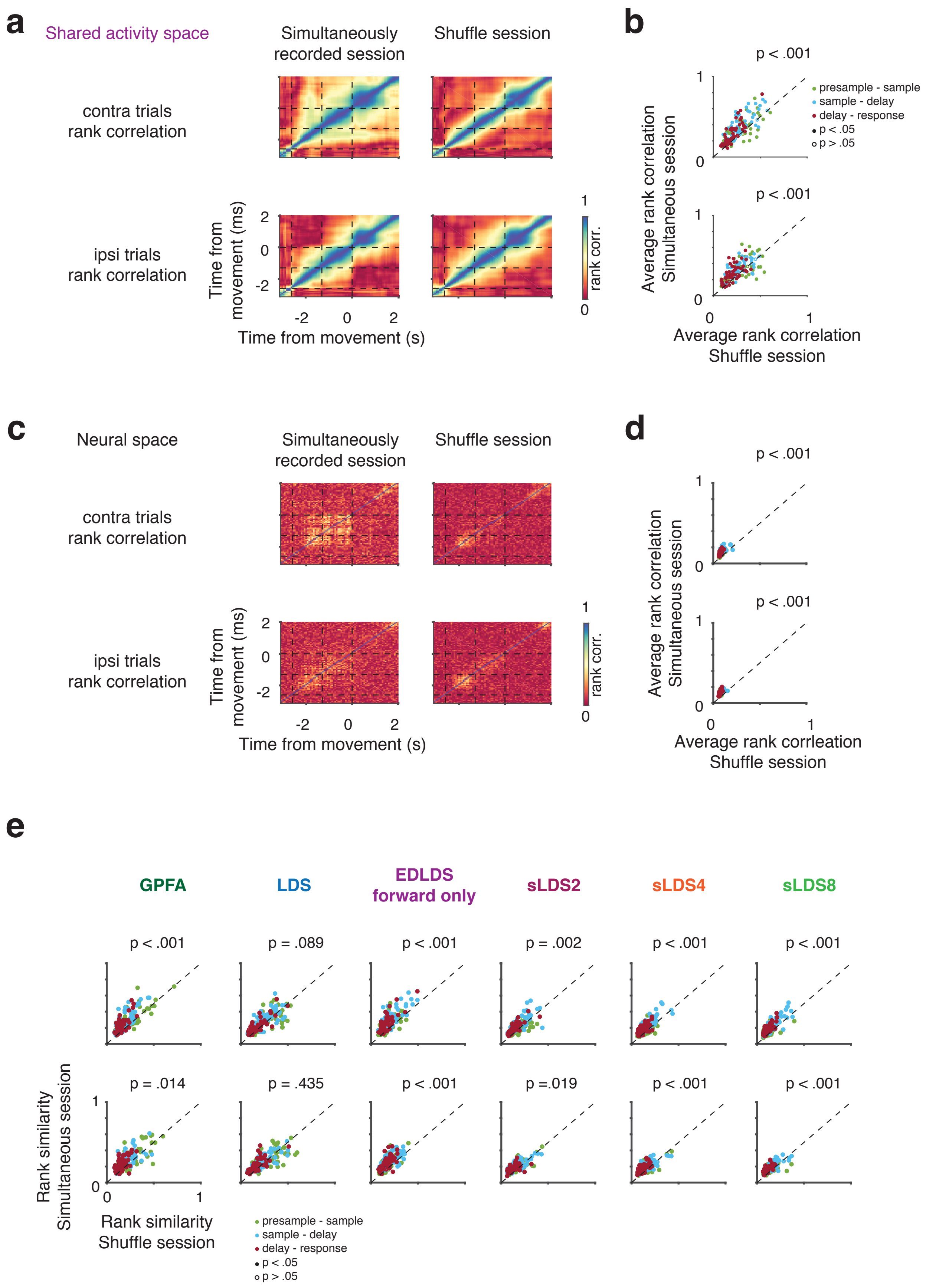

a
Shared activity space
Simultaneously recorded session
Shuffle session
contra trials rank correlation
ipsi trials rank correlation
Time from movement (ms)
2
0
-2
-2
0
2
Time from movement (s)
1
rank corr.
0
b
p < .001
1
0
presample - sample
sample - delay
delay - response
p < .05
p > .05
p < .001
1
0
Average rank correlation Simultaneous session
0
1
Average rank correlation Shuffle session
c
Neural space
Simultaneously recorded session
Shuffle session
contra trials rank correlation
ipsi trials rank correlation
Time from movement (ms)
2
0
-2
-2
0
2
Time from movement (s)
1
rank corr.
0
d
p < .001
1
0
p < .001
1
0
Average rank correlation Simultaneous session
0
1
Average rank corrleation Shuffle session
e
GPFA
LDS
EDLDS forward only
sLDS2
sLDS4
sLDS8
p < .001
p = .089
p < .001
p = .002
p < .001
p < .001
p = .014
p = .435
p < .001
p =.019
p < .001
p < .001
1
0
0
1
Rank similarity Simultaneous session
Rank similarity Shuffle session
presample - sample
sample - delay
delay - response
p < .05
p > .05

**Supplementary Figure 7. Dynamics of trial identity are disrupted in shuffle trials**

**a**. Comparison of rank correlation in simultaneous-recorded trial with shuffle trial session (based on the data from the same session, Session #17). The rank correlation is strong across epochs in simultaneous trials and is weak in shuffled the trial. Top, contra-only trials; Bottom, ipsi-only trials. **b**. Comparison across all sessions (n = 55). **c-d**. The same convention as **a-b** except in full neural space. **e**. the same convention as that in **b** except for other models. P-value is reported using sign-rank test.

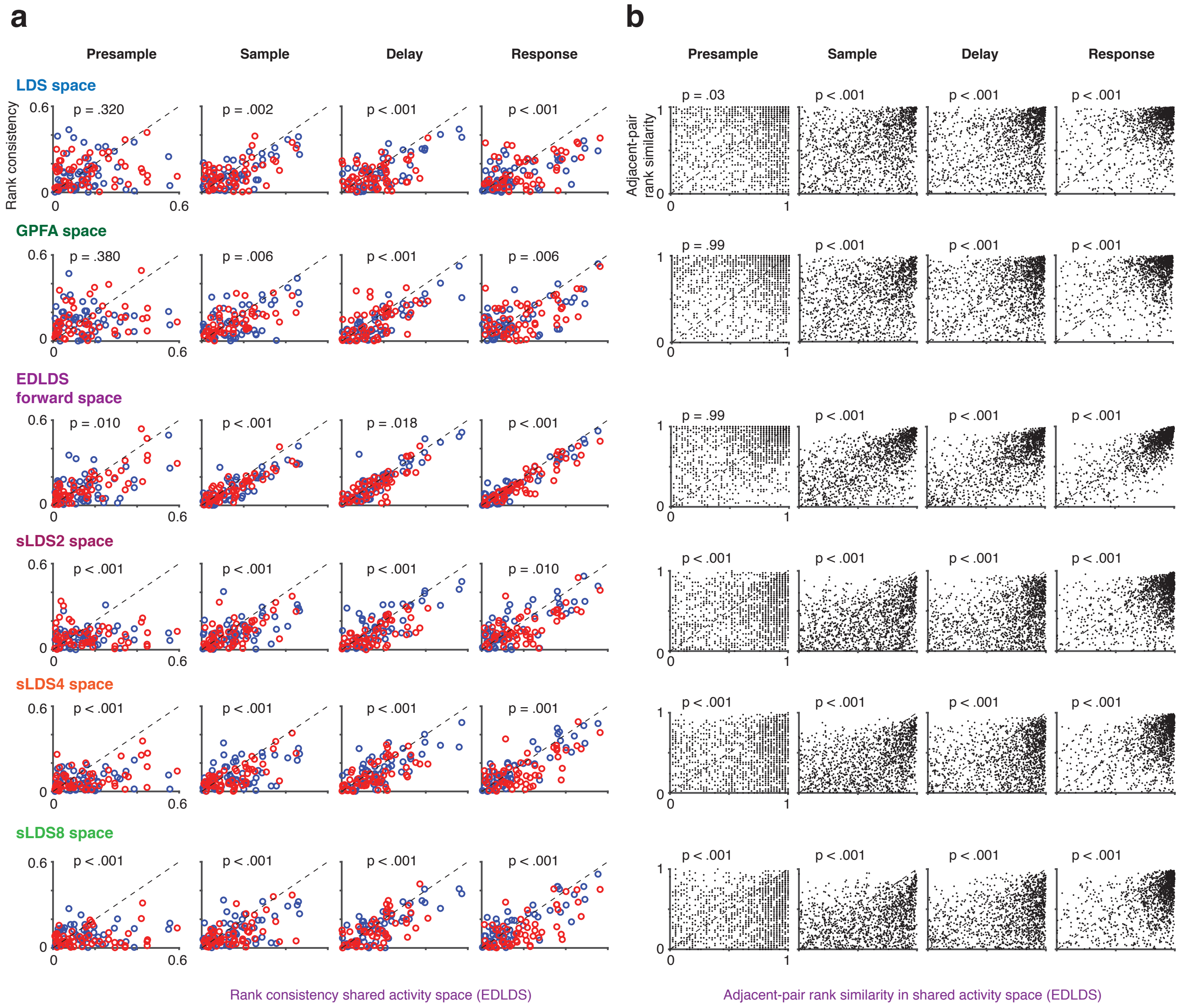


**Supplementary Figure 8. (Supporting figure for Figure 5cd)**

**a**. Following the same convention as that in **Figure 5c** except for other models. **b**. Following the same convention as that in **Figure 5d** except for other models. Both p-value is reported using sign-rank test.

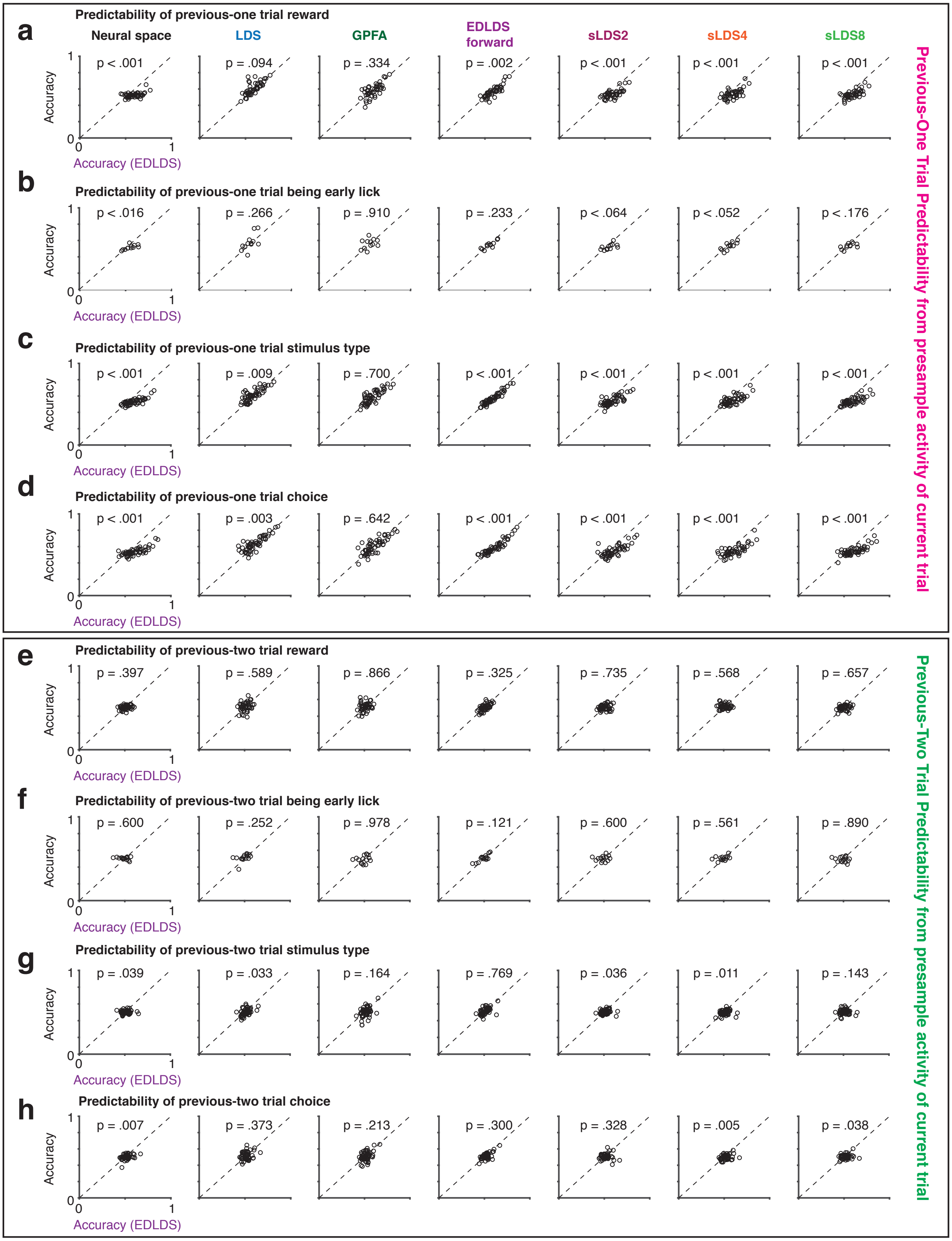


**Supplementary Figure 9. (Supporting figure for Figure 5fg)**

**a**. Following the same convention as that in **Figure 5f** except for all models. **b-d**. Following the same convention as that in **a.** except for predictions of previous trial being early lick, previous trial stimulus type and previous trial choice respectively. **e-h**. Following the same convention as that in **a-d** respectively except for predictions of previous-two trial. All p-value is reported using sign-rank test.

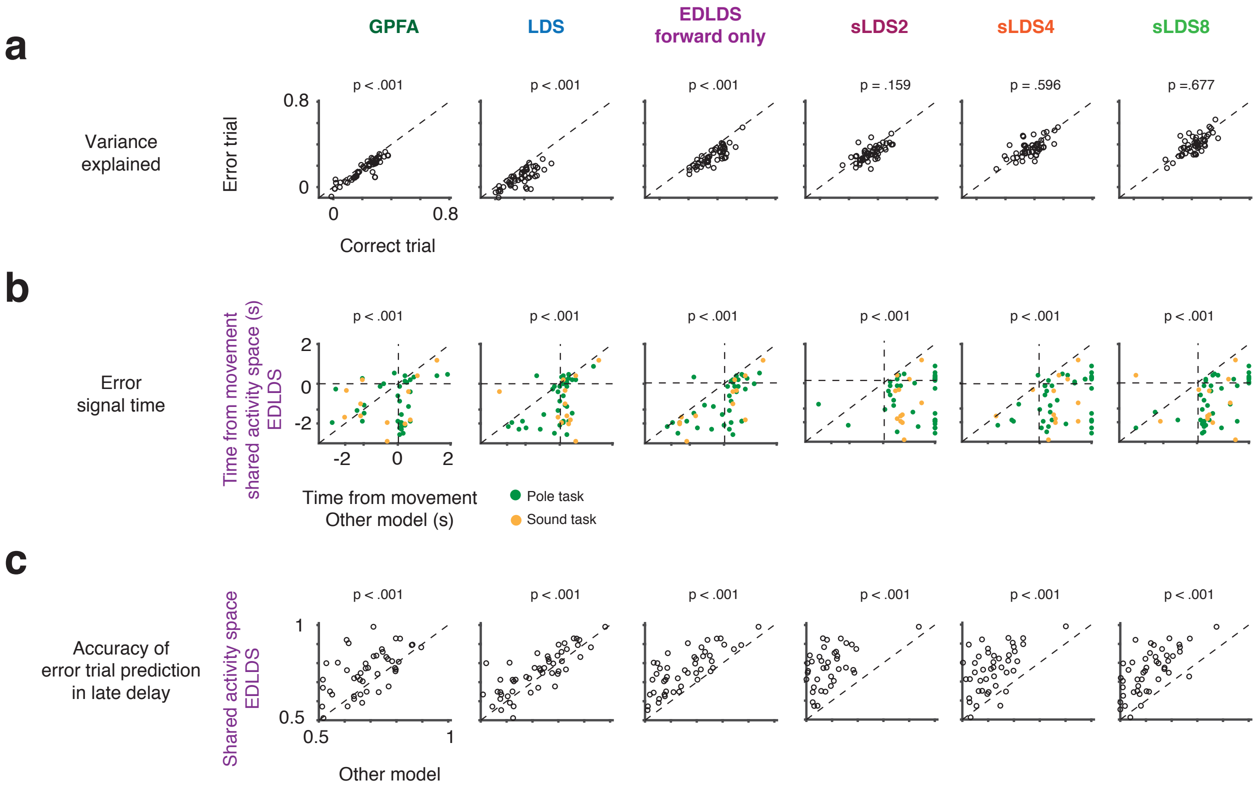


**Supplementary Figure 10. (Supporting figure for Figure 6bde)**

**a.** Following the same convention as that in **Figure 6b** except for other models. **b**. Following the same convention as that in **Figure 6d** except for other models. **c.** Following the same convention as that in **Figure 6e** except for other models. All p-value is reported using sign-rank test.

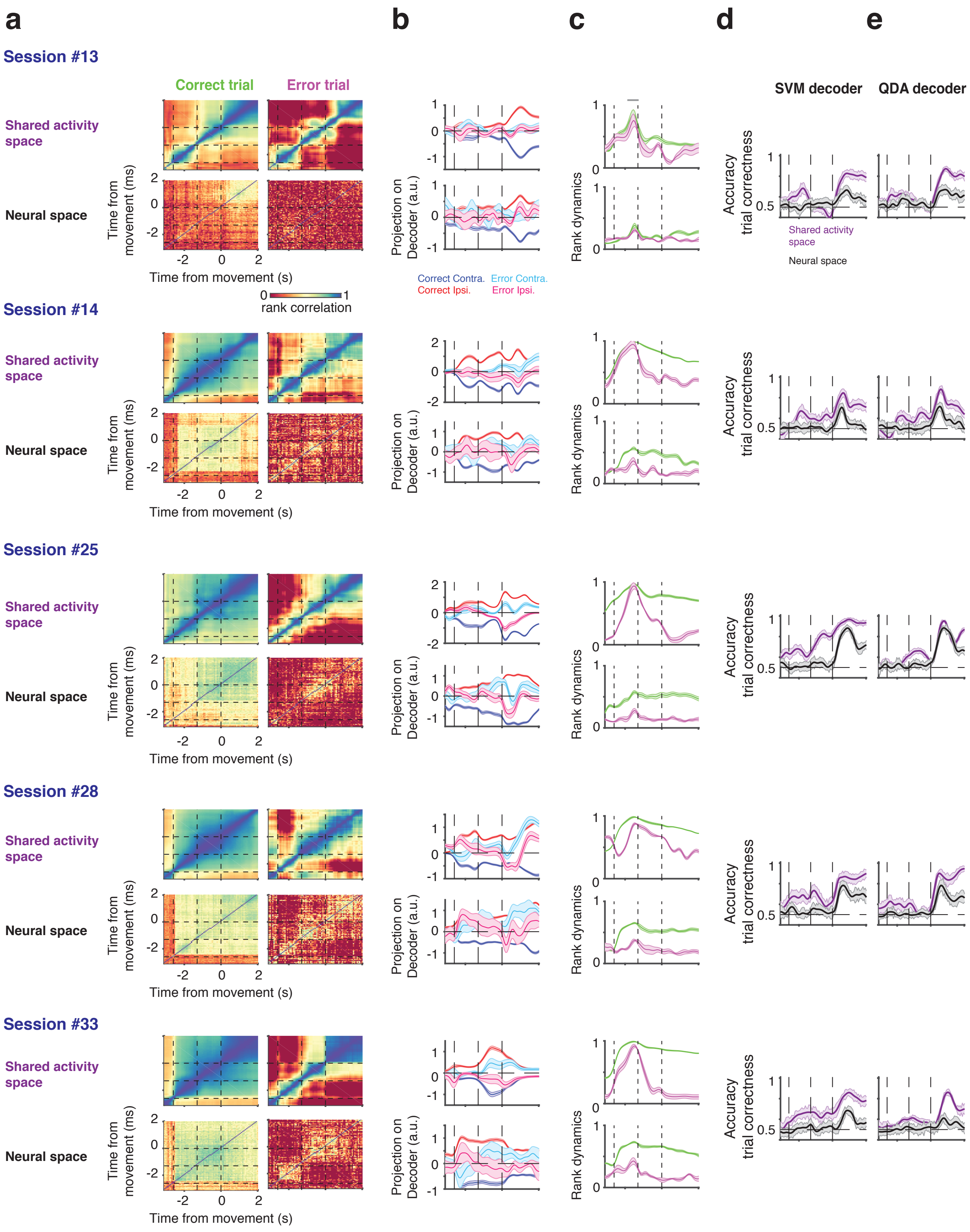


**Supplementary Figure 11. (Supporting figure for Figure 6cghi) Details of neural prediction of correctness on single trials for five more sessions**

**a.** the same convention as that in **Figure 6h**, except for five other sessions (Sessions #13, #14, #25, #28, #33). Top, based on neural rank dynamics in EDLDS shared activity space; bottom, based on that in full neural space. **b.** the same convention as that in **Figure 6g**, except for five other sessions. Top, based on neural rank dynamics in EDLDS shared activity space; bottom, based on that in full neural space. **c.** the same convention as that in **Figure 6i**, except for five other sessions. Top, based on neural rank dynamics in EDLDS shared activity space; bottom, based on that in full neural space. **d.** the same convention as that in **Figure 6c**, except for five other sessions. **e.** the same convention as that in **d.**, except using quadratic discriminant analysis to decode error signals.

## Supplementary Table 1. Data Summary

| Session Index | Task | # units | Contra. Correct # trials | Contra. Correct Spike range (Hz) | Ipsi. Correct # trials | Ipsi. Correct Spike range (Hz) | Contra. Error # trials | Contra. Error Spike range (Hz) | Ipsi. Error # trials | Ipsi. Error Spike range (Hz) | Recording | Depth range (um) min | Depth range (um) max | Cell type # pyr | Cell type # int | Latent dimensionality | Effective eigvalue Presample | Effective eigvalue Sample | Effective eigvalue Delay | Effective eigvalue Response |
|---|---|---|---|---|---|---|---|---|---|---|---|---|---|---|---|---|---|---|---|---|
| #01 | Pole | 6 | 23 | 4.47 - 13.16 | 24 | 5.32 - 11.44 | 4 | 5.20 - 16.86 | 5 | 4.09 - 15.61 | 32-channel | 274.036502 | 701.102059 | 4 | 2 | 2 | 0.85867265 | 0.98427532 | 0.98273796 | 0.97100392 |
| #02 | Pole | 7 | 45 | 5.89 - 25.25 | 29 | 4.83 - 27.83 | 15 | 5.72 - 26.03 | 6 | 4.53 - 25.02 | 32-channel | 390.411866 | 710.711034 | 5 | 2 | 3 | 0.90319136 | 0.98167062 | 1.00470606 | 0.98481919 |
| #03 | Pole | 8 | 32 | 3.11 - 25.08 | 29 | 3.46 - 24.76 | 8 | 3.54 - 24.11 | 3 | 4.18 - 28.72 | 32-channel | 370.126252 | 903.958199 | 7 | 1 | 4 | 0.90122289 | 1.00804556 | 1.00633234 | 1.0018386 |
| #04 | Pole | 7 | 22 | 4.06 - 19.47 | 22 | 4.17 - 18.32 | 7 | 4.21 - 20.07 | 5 | 3.31 - 17.46 | 32-channel | 293.254452 | 933.852788 | 6 | 1 | 2 | 0.86639202 | 0.9304176 | 0.92400783 | 0.99682407 |
| #05 | Pole | 11 | 34 | 9.31 - 40.76 | 33 | 2.66 - 35.01 | 7 | 4.54 - 41.46 | 4 | 7.81 - 41.77 | 32-channel | 371.193916 | 905.025863 | 8 | 3 | 4 | 0.94056857 | 1.00863458 | 0.98841162 | 0.94469153 |
| #06 | Pole | 6 | 39 | 5.59 - 15.59 | 53 | 5.51 - 17.73 | 20 | 4.99 - 18.85 | 5 | 4.47 - 18.54 | 32-channel | 489.704608 | 810.003776 | 4 | 2 | 2 | 0.76898586 | 0.85920438 | 0.98994183 | 0.89615163 |
| #07 | Pole | 7 | 38 | 5.74 - 23.21 | 18 | 3.46 - 26.20 | 7 | 2.78 - 14.95 | 29 | 4.07 - 23.53 | 32-channel | 230.262282 | 657.327839 | 5 | 2 | 4 | 0.91023989 | 0.98053077 | 0.98545277 | 0.99600335 |
| #08 | Pole | 6 | 38 | 4.33 - 32.43 | 33 | 4.00 - 28.35 | 9 | 4.20 - 23.47 | 32 | 4.67 - 31.35 | 32-channel | 577.253047 | 897.552215 | 6 | 0 | 3 | 0.89789757 | 0.98196783 | 0.98736427 | 0.99981268 |
| #09 | Pole | 9 | 42 | 5.02 - 50.38 | 61 | 3.51 - 45.64 | 1 | 3.47 - 46.83 | 28 | 4.80 - 55.19 | 64-channel | NaN | NaN | 6 | 3 | 5 | 0.89347428 | 0.99709429 | 1.00769547 | 0.99457506 |
| #10 | Pole | 8 | 28 | 3.62 - 18.81 | 33 | 4.96 - 17.82 | 0 | NaN - NaN | 6 | 5.75 - 21.20 | 64-channel | NaN | NaN | 7 | 1 | 3 | 0.93903955 | 0.9586466 | 0.99616445 | 0.97853203 |
| #11 | Pole | 10 | 38 | 5.89 - 22.54 | 55 | 3.85 - 26.04 | 0 | NaN - NaN | 9 | 4.05 - 21.65 | 64-channel | NaN | NaN | 7 | 3 | 4 | 1.00871187 | 0.95189305 | 0.99151915 | 0.95866899 |
| #12 | Pole | 11 | 77 | 4.48 - 21.70 | 97 | 4.69 - 22.52 | 4 | 3.23 - 22.79 | 11 | 3.45 - 21.53 | 64-channel | NaN | NaN | 8 | 3 | 5 | 0.91621648 | 1.00499858 | 0.99880837 | 0.99346456 |
| #13 | Pole | 11 | 74 | 4.54 - 51.30 | 83 | 4.16 - 49.18 | 12 | 4.95 - 50.25 | 19 | 4.04 - 56.16 | 64-channel | NaN | NaN | 7 | 4 | 5 | 0.93887565 | 0.96222839 | 1.0243967 | 1.00066949 |
| #14 | Pole | 12 | 92 | 5.99 - 21.96 | 93 | 5.41 - 17.03 | 13 | 5.38 - 24.15 | 13 | 6.11 - 25.13 | 64-channel | NaN | NaN | 10 | 2 | 6 | 0.99639386 | 0.99910228 | 1.0174949 | 0.97334146 |
| #15 | Pole | 11 | 28 | 5.64 - 32.75 | 39 | 5.94 - 32.19 | 6 | 6.52 - 27.75 | 7 | 6.03 - 34.91 | 64-channel | NaN | NaN | 9 | 2 | 5 | 0.912267 | 1.00017747 | 1.01249232 | 0.97419361 |
| #16 | Pole | 8 | 41 | 3.85 - 21.35 | 47 | 3.98 - 21.26 | 5 | 3.47 - 24.17 | 3 | 4.56 - 21.97 | 64-channel | NaN | NaN | 7 | 1 | 5 | 0.94134013 | 0.9350024 | 1.02898431 | 0.96540427 |
| #17 | Pole | 18 | 74 | 5.13 - 43.02 | 100 | 2.33 - 30.85 | 7 | 4.02 - 32.41 | 12 | 4.27 - 33.94 | 64-channel | NaN | NaN | 14 | 4 | 4 | 0.9428217 | 1.02365539 | 1.00777546 | 0.98011615 |
| #18 | Pole | 8 | 69 | 6.63 - 37.16 | 78 | 6.83 - 32.24 | 7 | 8.23 - 37.47 | 18 | 8.01 - 33.82 | 64-channel | NaN | NaN | 5 | 3 | 4 | 0.83355581 | 0.97397822 | 1.00659071 | 0.99217207 |
| #19 | Pole | 6 | 86 | 4.95 - 17.58 | 63 | 6.03 - 18.92 | 14 | 5.74 - 17.98 | 6 | 5.81 - 16.03 | 64-channel | NaN | NaN | 5 | 1 | 3 | 0.93433041 | 1.02107517 | 1.00909567 | 0.99282516 |
| #20 | Pole | 13 | 45 | 4.93 - 64.88 | 55 | 4.37 - 58.21 | 4 | 3.04 - 61.14 | 9 | 2.89 - 54.61 | 64-channel | NaN | NaN | 7 | 6 | 3 | 0.90012635 | 0.93302189 | 0.98501047 | 0.95151666 |
| #21 | Pole | 7 | 34 | 6.48 - 22.78 | 53 | 6.29 - 17.47 | 5 | 6.55 - 22.55 | 6 | 4.37 - 24.80 | 64-channel | NaN | NaN | 5 | 2 | 4 | 0.89886705 | 0.97882922 | 0.96890097 | 0.99034921 |
| #22 | Pole | 8 | 72 | 5.31 - 30.11 | 72 | 4.57 - 20.14 | 5 | 3.01 - 33.92 | 21 | 5.30 - 22.32 | 64-channel | NaN | NaN | 6 | 2 | 6 | 0.9411863 | 0.99577481 | 1.00018126 | 0.96255571 |
| #23 | Pole | 7 | 36 | 0.65 - 12.80 | 49 | 0.38 - 12.58 | 5 | 0.42 - 13.80 | 19 | 0.38 - 10.81 | 64-channel | 219.960981 | 849.826583 | 6 | 1 | 3 | 0.86614629 | 0.90154743 | 0.91767561 | 0.96472866 |
| #24 | Pole | 8 | 76 | 1.10 - 13.74 | 23 | 0.53 - 11.67 | 15 | 0.63 - 16.50 | 35 | 0.80 - 14.87 | 64-channel | 265.481443 | 670.770998 | 7 | 1 | 3 | 0.89168456 | 0.98639013 | 0.98337906 | 0.98558758 |
| #25 | Pole | 15 | 51 | 0.79 - 24.97 | 67 | 0.92 - 17.22 | 13 | 1.04 - 18.43 | 26 | 0.98 - 20.70 | 64-channel | 297.168251 | 862.470685 | 15 | 0 | 4 | 0.86820121 | 0.97185811 | 0.99898836 | 1.01307641 |
| #26 | Pole | 10 | 40 | 0.47 - 24.99 | 33 | 0.50 - 26.99 | 9 | 0.41 - 25.12 | 10 | 0.40 - 22.92 | 64-channel | 369.466912 | 817.660578 | 8 | 2 | 3 | 0.89284523 | 0.95691276 | 0.9387302 | 0.95711896 |
| #27 | Pole | 12 | 36 | 0.60 - 18.88 | 32 | 0.62 - 17.19 | 3 | 0.45 - 17.09 | 10 | 0.83 - 19.12 | 64-channel | 434.480804 | 950.404343 | 10 | 2 | 3 | 0.87945303 | 0.98942231 | 0.99216865 | 0.94092924 |
| #28 | Pole | 17 | 133 | 0.87 - 18.86 | 98 | 0.71 - 15.24 | 10 | 0.56 - 15.88 | 11 | 0.91 - 14.42 | 64-channel | 573.064339 | 1008.76698 | 14 | 3 | 5 | 0.93655417 | 0.99625865 | 1.00347068 | 0.99078433 |
| #29 | Pole | 18 | 84 | 0.87 - 22.75 | 59 | 0.45 - 18.09 | 27 | 0.58 - 12.66 | 7 | 0.44 - 18.89 | 64-channel | 466.89187 | 977.703293 | 13 | 5 | 5 | 0.91699516 | 0.99779532 | 0.99395805 | 1.00402952 |
| #30 | Pole | 10 | 82 | 0.68 - 20.25 | 80 | 0.48 - 18.27 | 7 | 0.36 - 13.93 | 12 | 0.21 - 17.17 | 64-channel | 748.582982 | 1004.86442 | 7 | 3 | 4 | 0.89024814 | 0.98394789 | 1.00867713 | 0.99120677 |
| #31 | Pole | 12 | 117 | 0.90 - 35.06 | 70 | 0.55 - 31.76 | 2 | 0.48 - 31.41 | 4 | 0.24 - 35.32 | 64-channel | 442.586271 | 1043.89892 | 9 | 3 | 4 | 0.97709582 | 0.9688005 | 1.01076114 | 1.00599633 |
| #32 | Pole | 14 | 115 | 2.32 - 29.42 | 94 | 1.94 - 23.15 | 19 | 0.84 - 27.60 | 5 | 1.43 - 32.42 | 64-channel | 489.181254 | 1030.5905 | 9 | 5 | 4 | 0.97446622 | 1.00173842 | 0.99051223 | 0.99702989 |
| #33 | Pole | 18 | 62 | 0.91 - 30.92 | 53 | 1.37 - 23.62 | 14 | 1.07 - 27.62 | 12 | 0.53 - 32.12 | 64-channel | 364.1958 | 1009.07623 | 13 | 5 | 4 | 0.91506911 | 1.00429121 | 1.0022627 | 1.01900086 |
| #34 | Pole | 18 | 76 | 0.66 - 21.97 | 69 | 0.63 - 25.53 | 16 | 0.83 - 28.55 | 6 | 0.58 - 35.56 | 64-channel | 327.596093 | 965.694331 | 16 | 2 | 5 | 0.93136978 | 1.01260166 | 0.98458819 | 1.00473779 |
| #35 | Pole | 11 | 92 | 0.83 - 41.53 | 69 | 0.60 - 39.86 | 4 | 1.49 - 38.26 | 15 | 0.81 - 41.23 | 64-channel | 317.942056 | 939.619524 | 7 | 4 | 4 | 0.90046645 | 0.98931682 | 0.99115983 | 0.95008305 |
| #36 | Pole | 12 | 97 | 0.73 - 26.40 | 96 | 0.59 - 16.91 | 11 | 0.54 - 16.57 | 11 | 0.58 - 22.44 | 64-channel | 459.638311 | 992.802568 | 11 | 1 | 4 | 0.93879437 | 0.98348036 | 1.01968206 | 0.99003434 |
| #37 | Pole | 22 | 118 | 0.65 - 57.25 | 87 | 0.61 - 31.00 | 10 | 0.66 - 28.52 | 8 | 1.04 - 36.35 | 64-channel | 509.989844 | 1006.42787 | 18 | 4 | 4 | 0.88100953 | 1.01105214 | 1.0049933 | 0.97869184 |
| #38 | Pole | 15 | 56 | 0.69 - 93.68 | 52 | 0.54 - 80.39 | 5 | 0.39 - 75.66 | 6 | 0.42 - 79.82 | 64-channel | 367.373358 | 1022.91108 | 7 | 8 | 4 | 0.88637604 | 0.99300474 | 0.99009268 | 0.97035636 |
| #39 | Pole | 16 | 61 | 0.36 - 49.61 | 54 | 0.51 - 44.61 | 8 | 0.53 - 45.29 | 3 | 0.32 - 38.67 | 64-channel | 380.483546 | 1013.98171 | 14 | 2 | 4 | 0.88360802 | 0.98903839 | 0.9934915 | 0.98268043 |
| #40 | Pole | 10 | 37 | 0.53 - 16.03 | 42 | 0.58 - 15.72 | 17 | 0.51 - 17.41 | 15 | 0.27 - 17.67 | 64-channel | 116.727134 | 395.577856 | 9 | 1 | 3 | 0.64206284 | 0.89602503 | 0.82614029 | 0.8818105 |
| #41 | Sound | 19 | 64 | 1.36 - 33.52 | 55 | 1.60 - 31.60 | 2 | 0.87 - 33.15 | 5 | 1.89 - 38.82 | 64-channel | 480.816905 | 953.33743 | 15 | 4 | 5 | 0.92649932 | 1.03668254 | 0.99930642 | 1.00185148 |
| #42 | Sound | 18 | 63 | 1.13 - 27.28 | 49 | 1.29 - 27.15 | 2 | 1.16 - 38.93 | 2 | 0.87 - 29.58 | 64-channel | 336.404497 | 1014.23985 | 14 | 4 | 6 | 0.9415931 | 1.01701986 | 0.99485981 | 1.00594766 |
| #43 | Sound | 11 | 100 | 1.92 - 28.33 | 56 | 0.88 - 17.03 | 4 | 0.96 - 20.67 | 3 | 1.16 - 20.17 | 64-channel | 534.733698 | 932.624984 | 11 | 0 | 4 | 0.87921918 | 0.98811316 | 1.00147062 | 0.96834797 |
| #44 | Sound | 23 | 70 | 2.05 - 30.77 | 58 | 1.22 - 30.38 | 14 | 0.99 - 34.20 | 6 | 1.77 - 39.61 | 64-channel | 374.315167 | 982.331405 | 17 | 6 | 6 | 0.87044244 | 0.98260544 | 1.00375229 | 0.98177374 |
| #45 | Sound | 16 | 95 | 1.83 - 19.10 | 77 | 1.23 - 8.99 | 11 | 1.12 - 14.17 | 14 | 1.10 - 19.30 | 64-channel | 514.412961 | 1059.37056 | 15 | 1 | 4 | 0.89893947 | 0.99157021 | 1.01034534 | 0.9951922 |
| #46 | Sound | 24 | 75 | 1.06 - 24.62 | 68 | 0.83 - 21.14 | 9 | 0.88 - 25.80 | 12 | 1.03 - 29.74 | 64-channel | 440.891938 | 1052.96262 | 20 | 4 | 6 | 0.86754168 | 0.98143217 | 0.99762099 | 0.98326064 |
| #47 | Sound | 20 | 53 | 1.17 - 33.01 | 38 | 0.94 - 31.12 | 1 | 0.00 - 41.24 | 5 | 1.12 - 38.82 | 64-channel | 480.816905 | 953.33743 | 16 | 4 | 4 | 0.90115202 | 1.02194528 | 1.01709082 | 1.00100905 |
| #48 | Sound | 19 | 29 | 0.78 - 26.92 | 47 | 1.32 - 27.11 | 2 | 1.16 - 38.93 | 1 | 0.00 - 26.02 | 64-channel | 336.404497 | 1014.23985 | 15 | 4 | 6 | 0.94537721 | 1.02716938 | 0.98692139 | 0.97325099 |
| #49 | Sound | 11 | 100 | 1.92 - 28.33 | 56 | 0.88 - 17.03 | 4 | 0.96 - 20.67 | 3 | 1.16 - 20.17 | 64-channel | 534.733698 | 932.624984 | 11 | 0 | 3 | 0.9046251 | 0.98990153 | 1.00280905 | 0.95846885 |
| #50 | Sound | 20 | 26 | 1.33 - 26.15 | 39 | 1.62 - 15.69 | 0 | NaN - NaN | 1 | 0.70 - 17.81 | 64-channel | 448.522199 | 1031.32071 | 18 | 2 | 8 | 0.92824321 | 1.00872928 | 0.99348589 | 0.99746797 |
| #51 | Sound | 31 | 70 | 0.93 - 36.88 | 39 | 1.24 - 34.70 | 4 | 1.06 - 31.51 | 4 | 1.30 - 32.04 | 64-channel | 267.696769 | 881.850476 | 27 | 4 | 8 | 0.8939905 | 0.98849677 | 1.00088472 | 0.96814888 |
| #52 | Sound | 27 | 74 | 1.06 - 46.01 | 32 | 0.54 - 31.41 | 1 | 0.19 - 37.00 | 2 | 0.48 - 37.87 | 64-channel | 445.547958 | 1017.25722 | 23 | 4 | 8 | 0.91656423 | 1.00385812 | 1.01868539 | 1.00703055 |
| #53 | Sound | 23 | 70 | 2.05 - 30.77 | 58 | 1.22 - 30.38 | 14 | 0.99 - 34.20 | 6 | 1.77 - 39.61 | 64-channel | 374.315167 | 982.331405 | 17 | 6 | 6 | 0.90788126 | 0.97317647 | 1.01053091 | 0.98134003 |
| #54 | Sound | 16 | 95 | 1.83 - 19.10 | 77 | 1.23 - 8.99 | 11 | 1.12 - 14.17 | 14 | 1.10 - 19.30 | 64-channel | 514.412961 | 1059.37056 | 15 | 1 | 6 | 0.88757079 | 1.00883879 | 0.98212964 | 0.97234825 |
| #55 | Sound | 25 | 75 | 1.06 - 24.62 | 44 | 0.74 - 21.69 | 6 | 0.67 - 25.70 | 11 | 1.02 - 29.84 | 64-channel | 440.891938 | 1052.96262 | 21 | 4 | 7 | 0.8844455 | 0.96496431 | 1.00180854 | 0.9747957 |

Session #01 - #08 available from public source: http://crcns.org/data-sets/motor-cortex/alm-1 ; Li et al., Nature, 2015; Nuo Li, Charles R Gerfen, Karel Svoboda, 2014.
Session #23 - #55 Inagaki et al., J. Neurosci., 2018.
All recompiled sessions available from public source: https://figshare.com/articles/Simultaneous_extracellular_electrophysiology_data/7372898/2

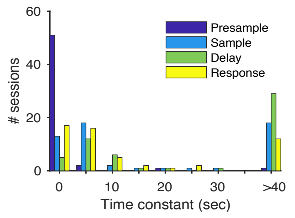

Supplement: Supplementary file 1 — Supplementary Information [file 41467_2018_8141_MOESM1_ESM.pdf]
