# Supplementary material for: An orderly single-trial organization of population dynamics in premotor cortex predicts behavioral variability
Source: Nat Commun. 2019 Jan 15;10:216. doi: 10.1038/s41467-018-08141-6 (PMC6333792; doi:10.1038/s41467-018-08141-6)
Supplement: Supplementary file 2 — Description of Additional Supplementary Files [file 41467_2018_8141_MOESM2_ESM.docx]

**Description of Additional Supplementary Files**

File Name: Supplementary Table 1

Description: Detailed session by session data and model fit properties. Our dataset is comprised of simultaneous population recording data from 55 sessions (downloadable at https://doi.org/10.6084/m9.figshare.7372898; 40 from a pole location discrimination task (Li et al., Nature, 2015; Inagaki., J. Neurosci., 2018; Wei et al., 2018); 15 from an auditory discrimination task (Inagaki., J. Neurosci.). For each session, we report (from left to right columns): the session index, task type, number of units, number of contra. correct trial, the spike range of units in contra. correct trials, number of ipsi. correct trial, the spike range of units in ipsi. correct trials, number of contra.error trial, the spike range of units in contra. error trials, number of ipsi. error trial, the spike range of units in ipsi. error trials, property of recording array, depth range of the units, number of the classified pyramidal cells, number of the classified interneurons, variance saturating dimension, and largest eigenvalues for pre-sample, sample, delay and responses in the fits (Supplementary Figure 2).
